# Haploinsufficiency for BRCA1 leads to cell-type-specific genomic instability and premature senescence

**DOI:** 10.1038/ncomms8505

**Published:** 2015-06-24

**Authors:** Maja Sedic, Adam Skibinski, Nelson Brown, Mercedes Gallardo, Peter Mulligan, Paula Martinez, Patricia J. Keller, Eugene Glover, Andrea L. Richardson, Janet Cowan, Amanda E. Toland, Krithika Ravichandran, Harold Riethman, Stephen P. Naber, Anders M. Näär, Maria A. Blasco, Philip W. Hinds, Charlotte Kuperwasser

**Affiliations:** 1 Program in Cell, Molecular and Developmental Biology, Sackler School of Graduate Biomedical Sciences, Tufts University School of Medicine, 136 Harrison Avenue, Boston, Massachusetts 02111, USA; 2 Molecular Oncology Research Institute, Tufts Medical Center, 800 Washington Street, Boston, Massachusetts 02111, USA; 3 Telomeres and Telomerase Group, Spanish National Cancer Centre, Madrid E-28029, Spain; 4 Department of Cell Biology, Harvard Medical School and Massachusetts General Hospital Cancer Center, Building 149, 13th Street, Charlestown, Massachusetts 02129, USA; 5 Department of Pathology, Harvard Medical School, Brigham and Women's Hospital, 75 Francis Street, Boston, Massachusetts 02115, USA; 6 Department of Pathology, Tufts Medical Center, 800 Washington Street, Boston, Massachusetts 02111, USA; 7 Department of Molecular Virology, Immunology, and Medical Genetics, Department of Internal Medicine's Division of Human Genetics, Ohio State University, Columbus, Ohio 43210, USA; 8 Molecular and Cellular Oncogenesis Program, The Wistar Institute, 36th and Spruce Sts. Philadelphia, Pennsylvania 19104, USA

## Abstract

Although BRCA1 function is essential for maintaining genomic integrity in all cell types, it is unclear why increased risk of cancer in individuals harbouring deleterious mutations in *BRCA1* is restricted to only a select few tissues. Here we show that human mammary epithelial cells (HMECs) from *BRCA1*-mutation carriers (*BRCA1*^mut/+^) exhibit increased genomic instability and rapid telomere erosion in the absence of tumour-suppressor loss. Furthermore, we uncover a novel form of haploinsufficiency-induced senescence (HIS) specific to epithelial cells, which is triggered by pRb pathway activation rather than p53 induction. HIS and telomere erosion in HMECs correlate with misregulation of SIRT1 leading to increased levels of acetylated pRb as well as acetylated H4K16 both globally and at telomeric regions. These results identify a novel form of cellular senescence and provide a potential molecular basis for the rapid cell- and tissue- specific predisposition of breast cancer development associated with BRCA1 haploinsufficiency.

## Abstract (web summary)

BRCA1 functions in all cell types to help preserve genomic stability but oncogenesis is restricted to only a few tissues. Here Sedic *et al.* demonstrate a novel *BRCA1* haploinsufficiency-induced senescence pathway in epithelial cells.

## Graphical abstract

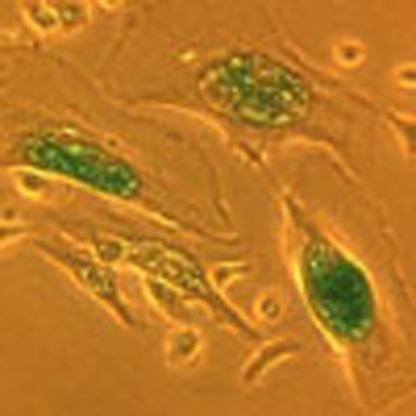

Inheriting one mutant copy of *BR*east *CA*ncer gene 1 (*BRCA1*) is linked to a significant increased risk of developing early-onset breast and ovarian cancer^1^,^2^. Breast tumours that develop in these individuals are different from sporadic cancers in that they arise rapidly (<6 mo) and often develop between mammographic screenings^3^. *BRCA1*-associated breast cancers are also unique in that they are generally more aggressive than sporadic breast cancers and are preferentially of the basal subtype^4^,^5^. Mouse models have been unable to reveal why mutations in a single *BRCA1* allele lead to increased and preferential risk in breast and ovarian cancer^6^,^7^. Thus, there may be fundamental species differences in the molecular circuitry linking BRCA1 function to cellular transformation that have not yet been defined.

BRCA1 is involved in an array of pathways essential for genomic maintenance such as homologous recombination, double-strand break repair, S-phase, G2/M and spindle checkpoints, as well as in centrosomal regulation^8^,^9^,^10^. Biallelic inactivation of BRCA1 leads to increased genomic instability and cancer development due to the essential role of this protein in coupling sensing and repairing of DNA damage to the cell-cycle machinery^8^,^9^,^10^. Notably, these proposed functions of BRCA1 have not been shown to be specific to breast epithelial cells. Thus, it remains unclear why *BRCA1* mutations are preferentially associated with increased incidence of cancer in only a small subset of tissues rather than a generalized increase in all cancer types, as is observed with other tumour-suppressor proteins involved in DNA damage repair (for example p53, ATM)^11^,^12^. In addition, for reasons that have remained obscure, it is unclear why *BRCA1*-mutation carriers exhibit an early and rapid onset of breast cancers^3^,^13^ when loss of the remaining wild-type (WT) *BRCA1* allele appears to be a late event during tumour progression^14^,^15^.

Inherited mutations in *BRCA1* lead to specific molecular and cellular alterations in breast epithelial differentiation before development of cancer; these changes are in part responsible for the propensity for basal-like tumour formation in *BRCA1*-associated breast cancers^16^,^17^. On the basis of this, we hypothesized that alterations in DNA damage response (DDR) pathways before the development of cancer might also be responsible for other phenotypes accompanying *BRCA1*-associated breast cancers: namely rapid tumour onset, extensive genomic instability and the preferential loss of p53 and pRb. In support of this, disease-free breast and ovarian tissues from *BRCA1*-mutation carriers have been shown to exhibit gene copy number gains and losses in key tumour suppressors and oncogenes^15^,^18^,^19^,^20^. In addition, deficiencies in error-free DNA damage repair have been observed in genetically engineered as well as primary *BRCA1*-haploinsufficient human mammary epithelial cells (HMEC) before *BRCA1* loss^18^,^19^,^20^,^21^.

Here we examine whether *BRCA1* haploinsufficiency is associated with cell-type or tissue-specific phenotypes in primary cells from disease-free breast and skin tissues of women with or without deleterious mutations in *BRCA1.* We report a unique cell-type-specific form of premature senescence associated with *BRCA1* haploinsufficiency as well as a molecular mechanism leading to rapid genomic instability in HMECs. This latter finding may explain in part the rapid onset of breast cancer development in individuals with *BRCA1* mutations.

## Results

### Increased DDR and genomic instability in BRCA1^mut/+^ HMECs

**Figure 1:**
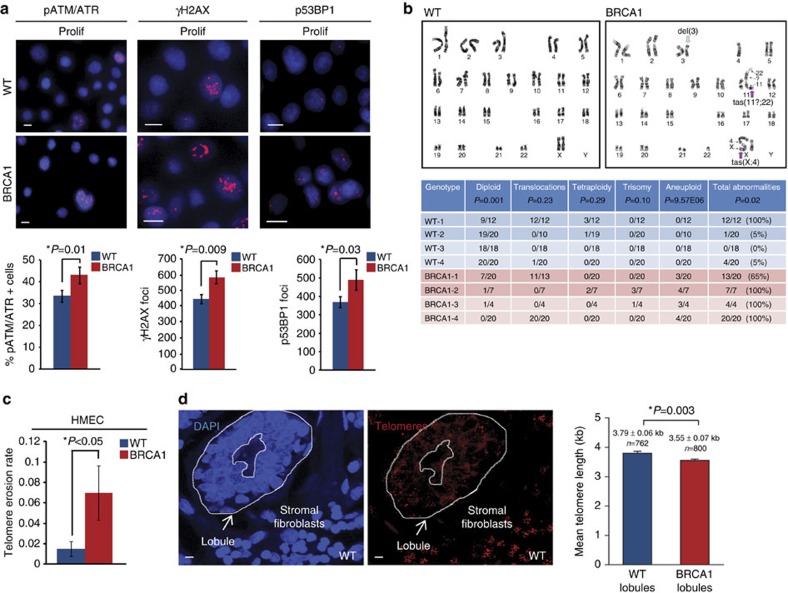
BRCA1^mut/+^ HMECs exhibit increased genomic instability and telomere dysfunction. (**a**) Representative images of immunofluorescence (IF) staining for phospho-ATM/ATR substrates, γH2AX foci, p53BP1 foci in proliferating (Prolif) WT and *BRCA1*^mut/+^ HMECs. Bar graphs under the images show the percent of cells positive for phospho-ATM/ATR substrates; the average number of γH2AX foci per field (avg number of cells per field WT=278, BRCA1=182); and the average number of p53BP1 foci per field (avg number of cells per field WT=262, BRCA1=187). (**b**) Representative images and summary table of significant genetic and chromosomal events determined by karyotype analysis in Prolif WT and *BRCA1*^mut/+^ HMECs. (**c**) Telomere erosion rate in WT (*n*=4) and *BRCA1*^mut/+^ (*n*=4) HMECs. Telomere length was determined by qPCR in Prolif and agonescent/senescent WT and *BRCA1*^mut/+^ HMECs. The telomere erosion rate was calculated by the formula: TER=Δtelomere length/ΔPDs. (**d**) Representative images and the mean telomere lengths (kb) determined using quantitative fluorescent *in situ* hybridization (qFISH) in WT lobules (*n*=762 cells) and *BRCA1*^mut/+^ lobules (*n*=800 cells). Student's two-tailed *t*-test was used to calculate *P* values. (*) indicates *P* value within the 0.05 level of significance. Error bar, s.e. Scale bar, 10 μm.

Induction of DDR involves activation of a molecular cascade leading to Ataxia telangiectasia mutated/Ataxia telangiectasia and Rad3-related (ATM/ATR) phosphorylation, kinase activation and phosphorylation of downstream substrates such as histone H2AX (γH2AX) at the site of DNA damage^22^. In addition, p53BP1 relocates to the sites of DNA damage where it becomes hyperphosphorylated because of ATM activation^23^. Given the recent evidence suggesting that *BRCA1* haploinsufficiency may be associated with increased DNA damage^15^,^18^,^19^,^20^,^21^, we examined the levels of DNA damage and activity of the DDR in WT and *BRCA1*^mut/+^ HMECs. The numbers of γH2AX and p53BP1 foci as well as the levels of substrates phosphorylated by ATM/ATR kinases were determined using immunofluorescence in proliferating cultures of WT and *BRCA1*^mut/+^ HMECs. *BRCA1*^mut/+^ HMECs exhibited significantly higher levels of phosphorylated ATM/ATR substrates as well as γH2AX and p53BP1 recruitment to DNA (*t*-test *P*=0.01; *P*=0.009; *P*=0.03, respectively; Fig. 1a) compared with WT cells. This was observed across multiple patient-derived *BRCA1*^mut/+^ HMECs and across multiple *BRCA1* mutations (Supplementary Table 1, BRCA1 expression level analysis in Supplementary Fig. 1), indicating that proliferating *BRCA1*^mut/+^ HMECs suffer increased DNA damage compared with WT cells.

To further corroborate these findings we compared the expression of genes involved in DDR regulation by gene set enrichment analysis (GSEA) in proliferating WT and *BRCA1*^mut/+^ HMECs. GSEA was applied to gene expression data collected on cultured proliferating primary HMECs isolated from *BRCA1*-mutation carriers (*N*=6) or age-matched WT patients (*N*=6; GSE19383; (ref. 24)). Consistent with increased DDR pathway activation, *BRCA1*^mut/+^ HMECs exhibited significant enrichment of genes associated with DNA repair (*t*-test *P*<0.0137; Supplementary Table 2), homologous recombination (*t*-test *P*<0.022; Supplementary Table 2) as well as genes involved in activation of ATR in response to replicative stress (*t*-test *P*<0.049; Supplementary Table 2).

Prolonged passaging and culture of primary WT HMECs (∼100 days, >20 population doublings (PDs)) leads to the accumulation of gross chromosomal abnormalities concomitant with telomere dysfunction, DDR and activation of the p53 signalling pathway^25^,^26^. Since *BRCA1*^mut/+^ HMECs displayed increased levels of DDR at early passages, we wanted to examine whether this might also be associated with a rapid accumulation of gross chromosomal abnormalities. Cytogenetic analysis of proliferating early-passaged WT and *BRCA1*^mut/+^ HMECs revealed that WT HMECs were mostly diploid with an occasional tetraploid cell (*t*-test *P*=0.001, Fig. 1b). Although most early-passaged WT HMECs did not exhibit significant chromosomal abnormalities, one sample (WT-1) had a single, identical translocation present in all cells most likely because of clonal expansion of this variant HMEC population. In contrast, early-passaged *BRCA1*^mut/+^ HMECs examined at the same PDs exhibited significant chromosomal abnormalities (*t*-test *P*<0.05, Fig. 1b). The majority of cells in several *BRCA1*^mut/+^ HMEC samples (BRCA1 and -4) exhibited frequent loss or gain of chromosomes as well as different types of chromosomal aberrations including unbalanced translocations and telomeric associations and fusions, which is indicative of telomeric dysfunction (Fig. 1b).

The increase in chromosomal alterations, particularly in lesions associated with telomere-end fusions, suggested that telomere dysfunction might be occurring in *BRCA1*^mut/+^ HMECs. To examine this, telomere length and telomere erosion rates (TERs) were measured in WT and *BRCA1*^mut/+^ HMECs. Quantitative PCR specific for telomere sequence was used to measure telomere length, and telomere erosion rates were calculated for each sample. Although early-passage WT and *BRCA1*^mut/+^ HMECs exhibited similar telomere lengths, late-passage *BRCA1*^mut/+^ HMECs displayed approximately fourfold increase in TER compared with late-passage WT HMECs (*t*-test *P*<0.04; Fig. 1c). These findings suggest that *BRCA1*^mut/+^ HMECs exhibit increased DNA damage and undergo rapid telomere dysfunction *in vitro*.

Since these *in vitro* findings may be a consequence of culture stress, we wanted to determine whether telomere erosion is also occurring *in vivo*. Telomere length was measured in disease-free human breast tissue using QFISH with a telomeric probe. Although telomere length was modestly shorter in luminal epithelial cells within lobules of *BRCA1*^mut/+^ breast tissues compared with those in WT breast tissues, this difference was statistically significant (Fig. 1d, *t*-test *P*=0.003). Moreover, luminal cells within lobules of WT (*N*=21) and *BRCA1*^mut/+^ (*N*=9) patients exhibited markedly reduced average telomere lengths as compared with epithelial cells from ducts and non-luminal breast epithelial cells in lobules (∼3.79 versus 6.65 kb in WT and ∼3.55 versus 6.57 kb BRCA1, Supplementary Fig. 2). This finding is of particular significance since the cellular precursors to breast cancers reside within lobules and luminal progenitor cells seem to be more privileged to increased telomere erosion than other breast epithelial cells^27^. Collectively, these findings show that *BRCA1*-haploinsufficient breast epithelial cells exhibit increased DDR, telomere dysfunction and genomic instability.

### BRCA1^mut/+^ HMECs undergo premature senescence

Cellular senescence is an intrinsic mechanism to suppress cellular proliferation and neoplastic transformation in many contexts including stress, telomere erosion, oncogene activation and most recently tumour-suppressor loss^28^,^29^,^30^. Since cultured *BRCA1*^mut/+^ HMECs exhibited increased telomere erosion and dysfunction, accompanied by increased DNA damage levels, we hypothesized that these cells might undergo premature senescence.

WT HMECs encounter two mechanistically distinct senescent-like barriers *in vitro* (Supplementary Fig. 3a,b)^25^,^31^,^32^,^33^,^34^. The first proliferative barrier, referred to as stasis or M0, is associated with classical p16/INK4a-dependent stress-induced senescence and concomitant p53 pathway activation (Supplementary Fig. 3a,c)^25^,^31^,^32^,^33^,^34^,^35^. Cells that emerge from this barrier do so through downregulation of p16/INK4a and rapidly proliferate until they reach the second proliferative barrier referred to as agonescence (Ag; Supplementary Fig. 3a,c)^25^,^34^. Unlike senescence, Ag is induced by p53 pathway activation in response to DNA damage and genomic instability as a consequence of telomere attrition and dysfunction^25^,^34^. In addition, the apparent proliferative arrest observed during Ag is maintained through a balance of proliferation and apoptosis^25^,^34^.

**Figure 2:**
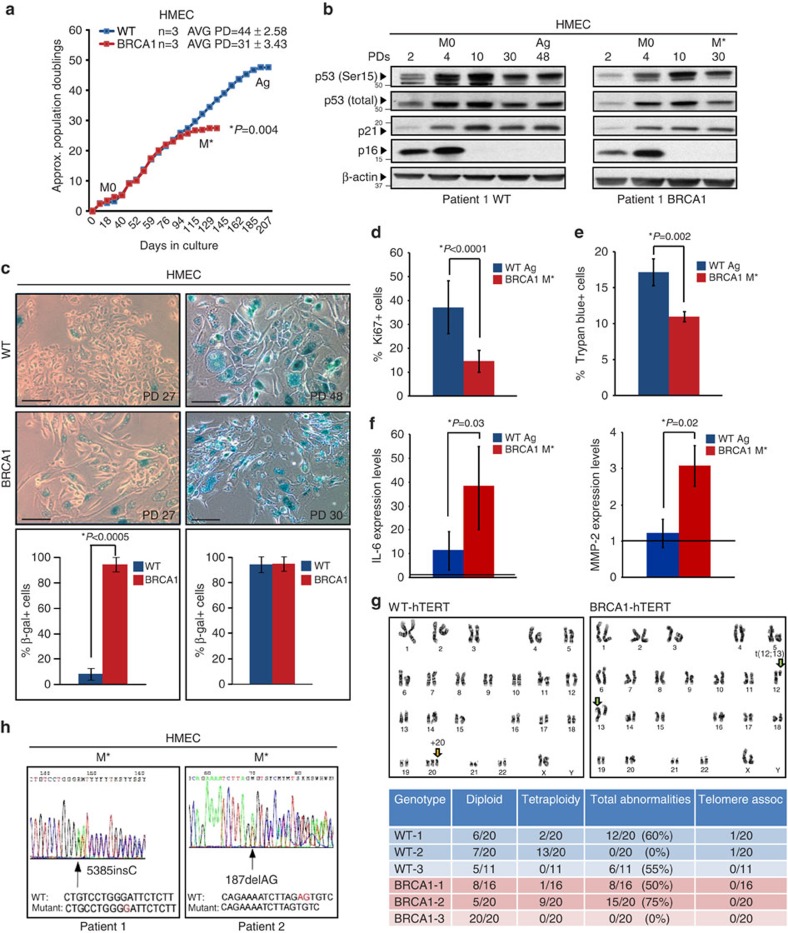
BRCA1^mut/+^ HMECs undergo premature senescence. (**a**) Representative growth curves of WT (*n*=3) and *BRCA1*^mut/+^(*n*=3) HMECs. (**b**) Western blot analysis of p16^INK4a^, total p53, p53 (Ser15) and p21 levels in WT and *BRCA1*^mut/+^ HMECs at indicated PDs. M0, stasis, Ag, agonescence (WT HMECs), M*, premature growth arrest (*BRCA1*^mut/+^ HMECs). (**c**) Brightfield images of SA-β-galactosidase staining and quantification of positive cells at selected PDs in WT and *BRCA1*^mut/+^ HMECs. (**d**) Percent of Ki-67-positive cells by IF staining in cultures of Ag WT and M* *BRCA1*^mut/+^ HMECs. (**e**) Percent of Trypan blue-positive cells in cultures of Ag WT and M* *BRCA1*^mut/+^ HMECs. (**f**) mRNA levels of IL-6 and MMP-2 (SASFs) in Ag WT and M* *BRCA1*^mut/+^ HMECs. The values were determined using qRT–PCR and normalized to proliferating cells (represented by line set at 1). (**g**) Representative images and summary table of significant genetic and chromosomal events determined by karyotype analysis in hTERT-expressing (immortalized) WT and *BRCA1*^mut/+^ HMECs. (**h**) LOH analysis in M* *BRCA1*^mut/+^ HMECs from two patient samples. Student's two-tailed *t*-test was used to calculate *P* values. (*) indicates *P* value within the 0.05 level of significance. Error bar, s.e. Scale bar, 100 μm.

Examination of *BRCA1*^mut/+^ and WT HMECs revealed similar growth kinetics and molecular responses in early cultures; both WT and *BRCA1*^mut/+^ HMECs entered into M0, induced p16/INK4a and p53 protein expression in a similar manner (Figs. 2a,b; Supplementary Fig. 3c). Likewise, WT and *BRCA1*^mut/+^ HMECs overcame M0 with similar frequencies and efficiencies, and both exhibited loss of p16/INK4a expression on emergence from stasis (Fig. 2a,b; Supplementary Fig. 3c). However, while WT HMECs on average continued to proliferate for an additional 44±2.58 STD PDs, *BRCA1*^mut/+^ HMECs stopped proliferating after 31±3.43 STD PDs (Fig. 2a). This premature growth arrest (M*) was observed across multiple patient-derived *BRCA1*^mut/+^ HMECs with different *BRCA1* mutations and was observed in *BRCA1*^mut/+^ HMECs well before Ag (*t*-test *P*=0.004, Supplementary Table 1). Cells in both M* and Ag displayed the senescent phenotype, characterized by enlarged, flattened morphology and positive staining for SA-β-galactosidase (Fig. 2c). However, unlike Ag, M* was characterized by significantly lower proliferation and apoptosis indexes indicating cell-cycle arrest (Fig. 2d,e; *t*-test *P*<0.0001, *P*=0.002, respectively; Supplementary Fig. 3d).

Senescence-associated secretory factors (SASFs) provide a molecular signature of senescence associated with severe DNA damage and help distinguish that from the cell-cycle arrest in the absence of DNA damage^36^,^37^. Examination of expression levels of SASFs such as interleukin (IL)-6, IL-8, matrix metalloproteinase (MMP)-2 and PAI-1 revealed that SASFs were not uniformly increased in M* *BRCA1*^mut/+^ HMECs compared with Ag WT HMECs (Fig. 2f, Supplementary Fig. 3e). Rather, only IL-6 and MMP-2, but not IL-8 or PAI-1, were increased. These findings combined with those above suggest that while premature senescence in *BRCA1*^mut/+^ HMECs is associated with severe DNA damage it is not identical to premature agonescence.

Since *BRCA1*^mut/+^ HMECs exhibited accelerated rate of telomere erosion as well as premature senescence, we hypothesized that activation of mechanisms that can stabilize telomere ends may be able to overcome this proliferative barrier and enhance genome stability. Indeed, overexpression of the catalytic subunit of human telomerase reverse transcriptase (hTERT) in *BRCA1*^mut/+^ HMECs resulted in telomere extension (*t*-test *P*=0.03, Supplementary Fig. 3f), enhanced genome stability (Fig. 2g) and immortalization. In addition, cytogenetic analysis revealed that the number of chromosomal rearrangements associated with telomere erosion (that is, telomeric associations) was attenuated in hTERT-expressing *BRCA1*^mut/+^ HMECs (Fig. 2g). Consistent with the above findings, these data indicate that rapid telomere dysfunction in *BRCA1*^mut/+^ HMECs is likely the trigger for premature senescence *in vitro*.

Loss of heterozygosity (LOH) of tumour-suppressor genes (for example, *VHL*, *PTEN*, *NF1* or *BRCA1*) can lead to the induction of premature senescence programmes^6^,^28^,^29^,^30^. LOH is frequently observed in *BRCA1*-associated cancers and in tissues of *BRCA1*-mutation carriers, indicating that *BRCA1*-haploinsufficient cells have increased propensity to lose the *BRCA1* allele^14^,^15^. Given that *BRCA1*^mut/+^ HMECs exhibited increased large-scale genomic instability, we examined whether premature senescence in these cells might be occurring because of LOH of the remaining WT *BRCA1* allele and decreased BRCA1 expression. PCR-based Sanger sequencing method was used to interrogate the individual *BRCA1*-mutation sites for LOH in *BRCA1*^mut/+^ HMECs. Interestingly, in both proliferating and senescent cells the WT allele was still retained (Fig. 2h, Supplementary Fig. 3g) indicating that premature senescence in *BRCA1*^mut/+^ HMECs is not through LOH. Furthermore, BRCA1 protein was expressed in *BRCA1*^mut/+^ HMECs, also confirming that LOH was not occurring (Supplementary Fig. 1). Thus, haploinsufficiency for *BRCA1* results in the engagement of a novel premature senescence-like barrier (a process hereafter termed: haploinsufficiency-induced senescence (HIS)).

### Premature senescence is cell-type-specific

**Figure 3:**
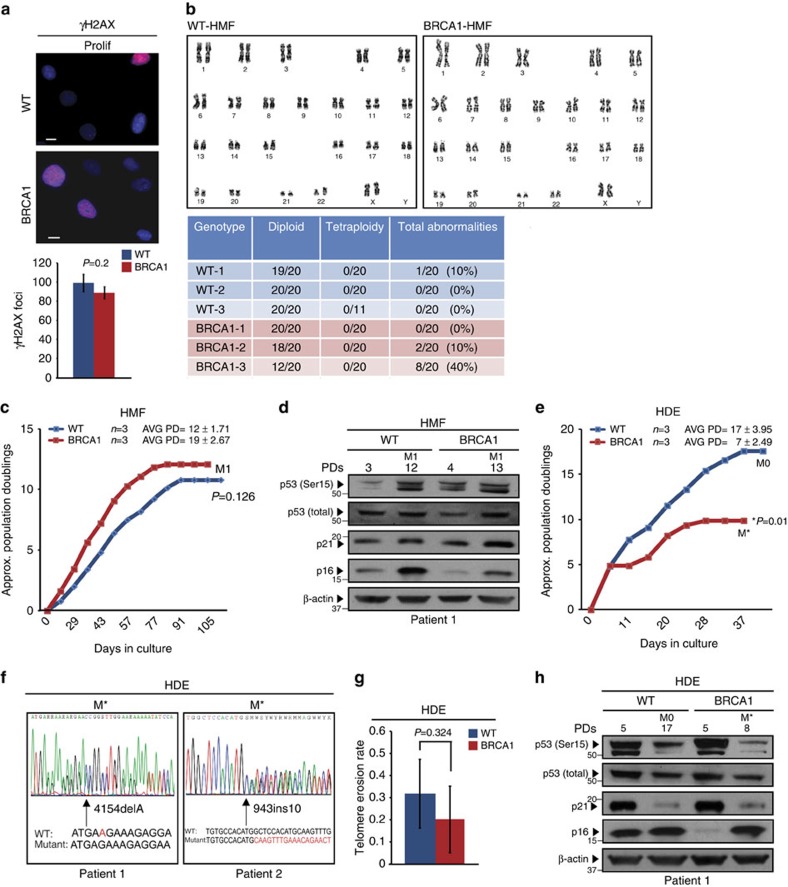
Premature senescence is cell-type-specific. (**a**) Representative images of IF staining for γH2AX foci in WT and *BRCA1*^mut/+^ HMFs on day 100 in culture (same day in culture as proliferating HMECs). Graph under the images shows the average number of γH2AX foci per field (avg number of cells per field WT=278, BRCA1=182). (**b**) Representative images and summary table of significant genetic and chromosomal events determined by karyotype analysis in WT and *BRCA1*^mut/+^ HMFs on day 100 in culture (same day in culture as proliferating HMECs). (**c**) Representative growth curves of WT (*n*=3) and *BRCA1*^mut/+^(*n*=3) HMFs. (**d**) Western blot analysis of p16^INK4a^, total p53, p53 (Ser15) and p21 levels in WT and *BRCA1*^mut/+^ HMFs at indicated PDs. M1, senescence. (**e**) Representative growth curves of WT (*n*=3) and *BRCA1*^mut/+^(*n*=3) HDEs. (**f**) LOH analysis in M* *BRCA1*^mut/+^ HDEs from two patient samples. (**g**) Telomere erosion rate in WT (*n*=3) and *BRCA1*^mut/+^ (*n*=3) HDEs. (**h**) Western blot analysis of p16^INK4a^, total p53, p53 (Ser15) and p21 levels in WT and *BRCA1*^mut/+^ HDEs at indicated PDs. M0, stasis. Student's two-tailed *t*-test was used to calculate *P* values. (*) indicates *P* value within the 0.05 level of significance. Error bar, s.e. Scale bar, 10 μm.

To determine whether BRCA1-associated HIS, DDR and genomic instabilities were unique to cultured HMECs, fibroblasts isolated from disease-free breast (human mammary fibroblasts (HMF)) and skin (human dermal fibroblasts (HDF)) tissues of women with or without deleterious mutations in *BRCA1* were examined (Supplementary Table 1, BRCA1 expression level analysis in Supplementary Fig. 1). Inspection of γH2AX foci formation and chromosomal abnormalities revealed that proliferating WT and *BRCA1*^mut/+^ HMFs exhibited similar numbers of γH2AX foci per nucleus (Fig. 3a) as well as few chromosomal rearrangements of no significant difference (Fig. 3b). Senescence was also evaluated in WT and *BRCA1*^mut/+^ mammary and skin fibroblasts; both WT and *BRCA1*^mut/+^ cells underwent comparable PDs in culture, after which cells became SA-β-galactosidase-positive and stopped dividing (Fig. 3c; Supplementary Fig. 4a,b). In addition, HMF and HDF exhibited a similar induction of p53 and p16/INK4a as they approached senescence (Fig. 3d; Supplementary Fig. 4c,d). These findings suggest that HIS, increased DDR and genomic instability are cell-type-specific.

Since *BRCA1*^mut/+^ fibroblasts did not exhibit premature senescence, we next examined whether this proliferative barrier was induced in other epithelial cell types. Primary keratinocytes (HDEs) were isolated from age-matched WT and *BRCA1*-mutation carriers and also examined for senescence. Similar to *BRCA1*^mut/+^ HMECs, premature growth arrest was observed in *BRCA1*^mut/+^ HDEs with typical features of senescence (Avg PD=7±2.5 versus Avg PD=17±4, respectively; *t*-test *P*=0.01, Fig. 3e). In addition, premature senescence in *BRCA1*^mut/+^ HDEs occurred without loss of the remaining WT allele indicating that the premature growth arrest was HIS (Fig. 3f). However, unlike *BRCA1*^mut/+^ HMECs, telomere lengths and erosion rates did not differ between WT and *BRCA1*^mut/+^ HDEs (*t*-test *P*=0.324, Fig. 3g) suggesting that HIS in HDEs was not associated with telomere dysfunction.

### HIS is mediated by active pRb signalling pathway

**Figure 4:**
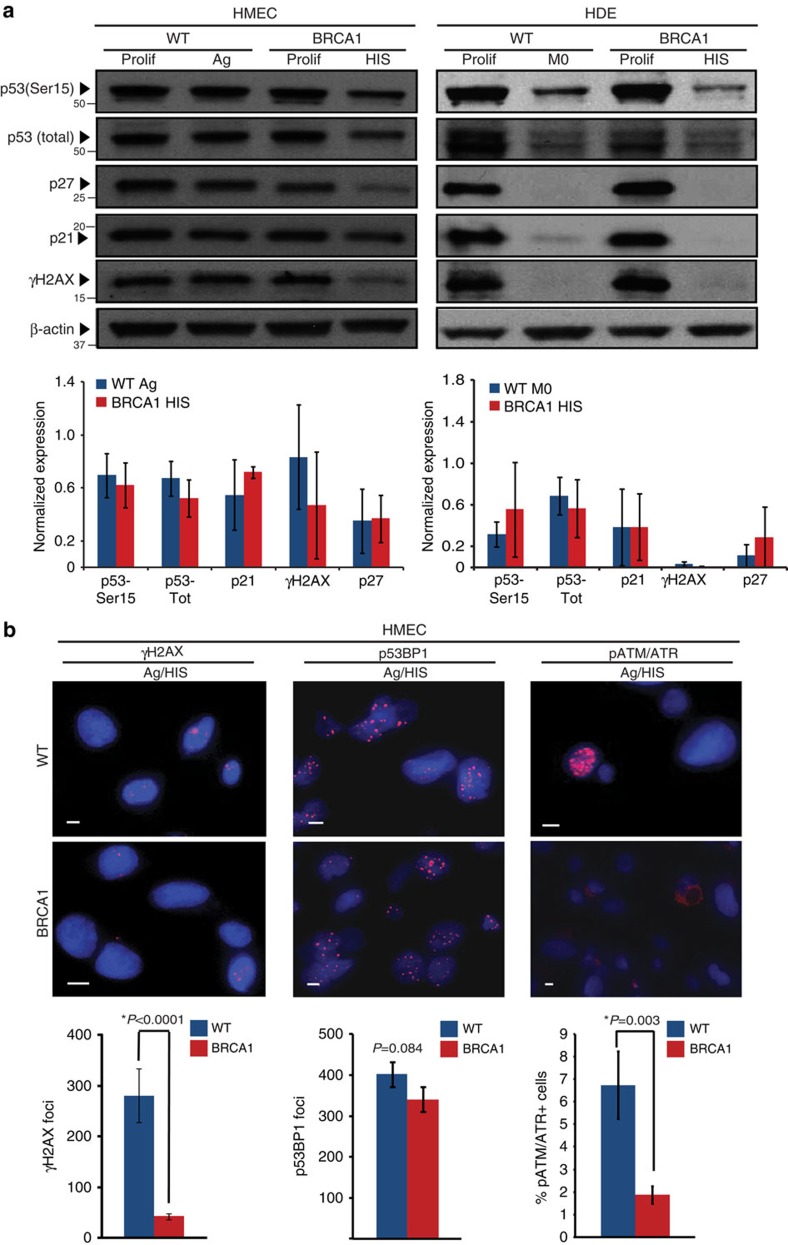
HIS does not correlate with increased p53 signalling pathway activation. (**a**) Western blot analysis of p53 (Ser15), total p53, γH2AX, p21 and p27 levels in Prolif and agonescent/HIS (Ag/HIS) WT and *BRCA1*^mut/+^ HMECs and Prolif and M0/HIS WT and *BRCA1*^mut/+^ HDEs. Bar graphs bellow the blots show the average levels of p53 (Ser15), total p53, γH2AX, p21 and p27 levels determined using western blot analysis in Ag WT (*n*=3) and HIS *BRCA1*^mut/+^ (*n*=3) HMECs and M0 WT (*n*=3) and HIS *BRCA1*^mut/+^ (*n*=3) HDEs. (**b**) Representative images of IF staining for phospho-ATM/ATR substrates, γH2AX foci, p53BP1 foci in Ag WT and HIS *BRCA1*^mut/+^ HMECs. Bar graphs under the images show the percent of cells positive for phospho-ATM/ATR substrates, the average number of γH2AX foci per field (avg number of cells per field WT=51, BRCA1=78) as well as the number of p53BP1 foci per field (avg number of cells per field WT=79, BRCA1=99). Student's two-tailed *t*-test was used to calculate *P* values. (*) indicates *P* value within the 0.05 level of significance. Error bar, s.e. Scale bar, 10 μm.

**Figure 5:**
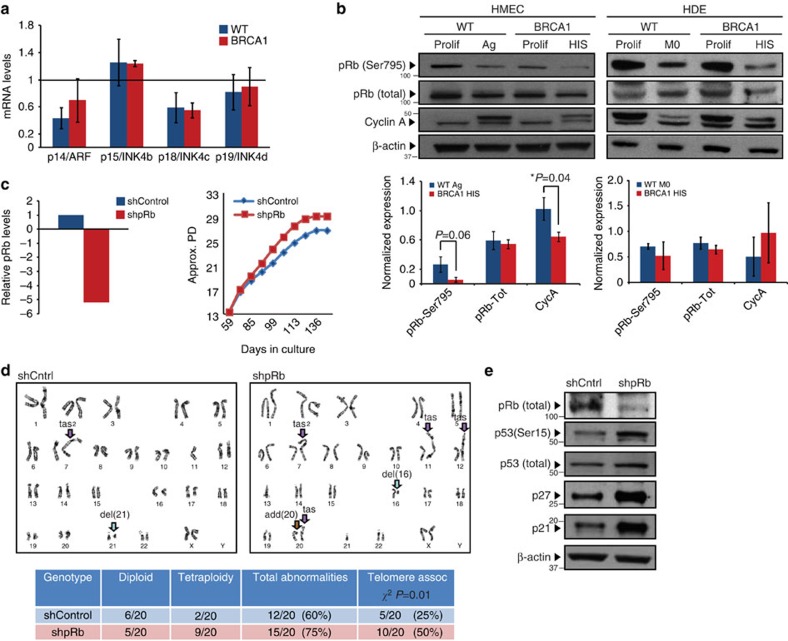
The active pRb signalling pathway mediates HIS. (**a**) mRNA levels of p14/ARF, p15/INK4b, p18/INK4c and p19/INK4d in Ag WT and HIS *BRCA1*^mut/+^ HMECs. The values were determined using qRT–PCR and normalized to proliferating cells (represented by line set at 1). (**b**) Western blot analysis of phospho-pRb (Ser795), total pRb and Cyclin A levels in Prolif and Ag/HIS WT and *BRCA1*^mut/+^ HMECs and Prolif and M0/HIS WT and *BRCA1*^mut/+^ HDEs. Bar graphs bellow the blots show the average levels of phospho-pRb (Ser795), total pRb and Cyclin A levels determined using western blot analysis in Ag WT (*n*=3) and HIS *BRCA1*^mut/+^ (*n*=3) HMECs and M0 WT (*n*=3) and HIS *BRCA1*^mut/+^ (*n*=3) HDEs. (**c**) pRb knockdown in proliferating *BRCA1*^mut/+^ HMECs (mRNA levels) and growth curve of control (shScr) and shpRb *BRCA1*^mut/+^ HMECs. (**d**) Representative images and summary table of significant genetic and chromosomal events determined by karyotype analysis in proliferating shScr (control) and shpRb *BRCA1*^mut/+^ HMECs. (**e**) Western blot analysis of pRb, p53 (Ser15), total p53, p21 and p27 levels in growth-arrested shScr (control) and shpRb *BRCA1*^mut/+^ HMECs. Student's two-tailed *t*- and *χ*^2^-tests were used to calculate *P* values. (*) indicates *P* value within the 0.05 level of significance. Error bar, s.e.

P53 is the major inducer of senescence in response to DNA damage and telomere dysfunction^38^ and is the primary inducer of premature senescence in *BRCA1*-deficient cells^6^. Therefore, we examined p53 activity and signalling pathway in senescent *BRCA1*^mut/+^ HMECs and HDEs. The levels of critical components of DDR and p53 pathway activation, such as phosphorylated p53 (Ser15), total p53, p21, p27, p14/ARF as well as phosphorylated ATM/ATR substrates, γH2AX and p53BP1, were not elevated in *BRCA1*^mut/+^ HMECs or HDEs indicating that there was no preferential induction of the p53 pathway in *BRCA1* heterozygous cells leading to HIS (Fig. 4a,b; Fig. 5a, Supplementary Figs 5a and 6a,b). Furthermore, the number of cells with phosphorylated ATM/ATR substrates (*t*-test *P*=0.003) and γH2AX foci (*t*-test *P*<0.0001) were actually reduced in senescent *BRCA1*^mut/+^ HMECs compared with agonescent WT HMECs (Fig. 4b), which is consistent with attenuated p53 pathway activation in these cells.

Since activation of the p53 signalling pathway and DDR were not increased at senescence (Fig. 4b)^21^, we next examined whether activation of the pRB signalling pathway might be the primary regluator of cell-cycle arrest during HIS. Premature senescence can be induced in the context of increased expression of various cyclin-dependent kinase inhibitors such as p16/INK4a, p15/INK4b, p18/INK4c and p19/INK4d. *BRCA1*^mut/+^ HDEs exhibited robust p16/INK4a protein induction on senescence (Fig. 3h). However, *BRCA1*^mut/+^ HMECs did not exhibit preferential induction of p16/INK4a expression nor did the levels of p15/INK4b, p18/INK4c and p19/INK4d differ between WT and *BRCA1*^mut/+^ HMECs to support the role of these factors in induction of HIS (Figs 2b and 5a; Supplementary Fig. 3c).

Next, we assesed the levels of pRb phosphorylation and E2F target genes (cyclin A and cyclin E) in HMECs and HDEs during HIS. Although total levels of pRb were similar, levels of phosphorylated pRb at Ser795 were reduced in senescent *BRCA1*^mut/+^ HMECs compared with WT HMECs (Fig. 5b, Supplementary Fig. 5b). In addition, levels of cyclin A were significantly decreased in senescent *BRCA1*^mut/+^ HMECs compared with WT HMECs (Fig. 5b, Supplementary Fig. 5b,c). In addition, senescence in HDEs was also associated with decreased levels of pRb phosphorylation and cyclin A expression (Fig. 5b, Supplementary Fig. 6c). Consistent with these data, GSEA of gene expression data from *BRCA1*^mut/+^ HMECs also revealed a significant enrichment of various pRb target genes, including those associated with senescence (*t*-test *P*<10^4^; Supplementary Table 2), E2F1-regulated genes (*t*-test *P*<10^4^; Supplementary Table 2) as well as genes downregulated in senescent cells lacking p53 activity (*t*-test *P*<10^4^; Supplementary Table 2). Altogether, these results suggest that HIS in epithelial cells is associated with pRb pathway activation.

To determine whether pRb was the primary inducer of HIS, lentiviral-mediated short hairpins were used to inhibit pRb expression (shpRb). Compared with control, knockdown of pRb in *BRCA1*^mut/+^ HMECs led to an increase in replicative potential (Fig. 5c, Supplementary Fig. 5d), indicating that pRb activity was mediating premature senescence. Moreover, cytogenetic analysis of *BRCA1*^mut/+^ HMECs forced to proliferate as a result of pRb knockdown revealed further increase in genomic instability associated with telomere-end fusions (*χ*^2^ *P*=0.01, Fig. 5d). Interestingly, shpRb *BRCA1*^mut/+^ HMECs did eventually undergo growth arrest (Fig. 5c, Supplementary Fig. 5d). We found that this proliferative barrier was associated with elevated levels of all components of the p53 signalling pathway (phosphorylated p53 (Ser15), total p53, p21, p27; Fig. 5e). Collectively, these data indicate that activation of the pRb pathway is the primary mediator of HIS in *BRCA1*^mut/+^ epithelial cells, and when bypass of HIS is forced (via downregulation of pRb), it results in the activation of the p53 pathway and accumulation of additional genomic abnormalities.

### SIRT1 regulates HIS in BRCA1^mut/+^ HMECs

Given the lack of p16/INK4a induction in *BRCA1*^mut/+^ HMECs despite pRb activation, we speculated that an alternate mechanism must be responsible for activating pRb in these cells. Indeed, pRb phosphorylation on multiple residues can be regulated by acetylation events catalysed by the NAD-dependent deacetylase SIRT1 in pRb–SIRT1 complexes^39^. In addition, it has been shown that SIRT1 protein expression decreases during replicative senescence and that there is a negative correlation between levels of SIRT1 and SA-β-galactosidase activity^40^,^41^. The cell-cycle arrest in these settings is associated with both decreased pRb phosphorylation and increased pRb acetylation^41^. In addition, SIRT1 also deacetylates histone H3K9, H3K56 and H4K16 during cellular aging on telomeric and subtelomeric regions, leading to loss of histones, shorter telomeres and genomic instability^42^,^43^. Thus, we reasoned that misregulation of SIRT1 in *BRCA1*^mut/+^ HMECs might result in both modifications of pRb acetylation leading to induction of HIS and changes in histone acetylation resulting in telomere dysfunction and increased genomic instability.

**Figure 6:**
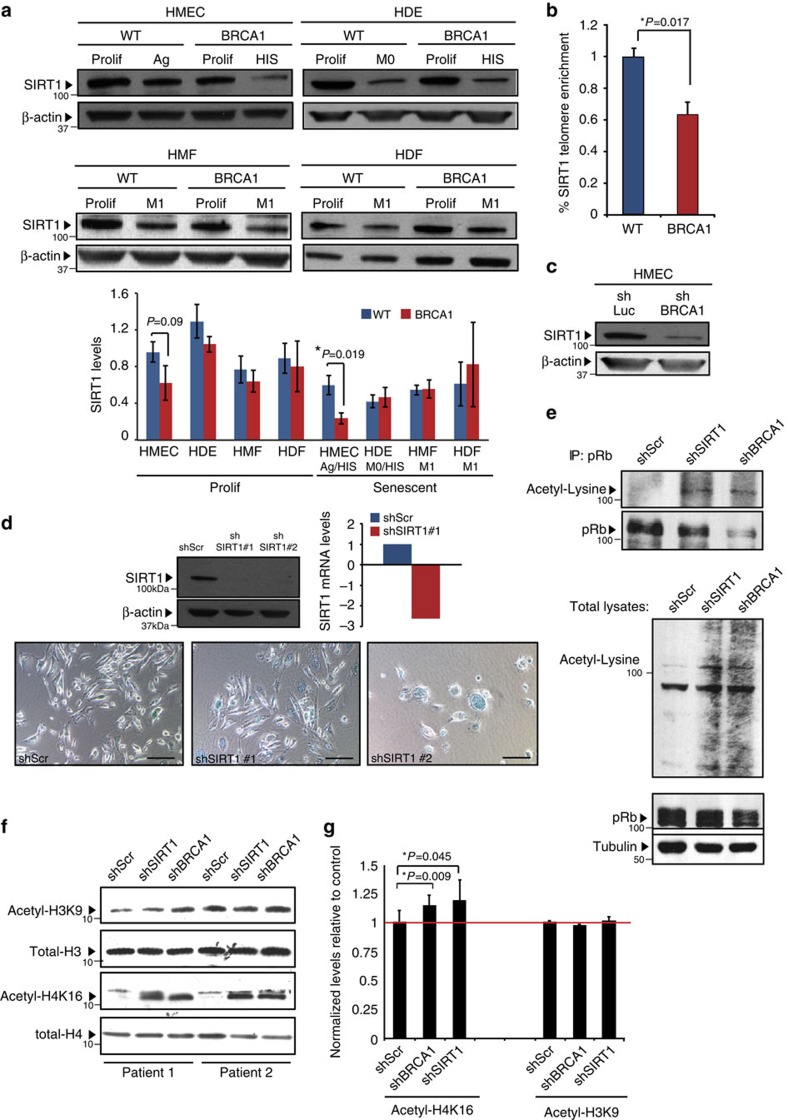
SIRT1 regulates HIS in BRCA1^mut/+^ HMECs. (**a**) Western blot analysis and quantification of SIRT1 levels in Prolif and Ag/HIS WT (*n*=3) and *BRCA1*^mut/+^ (*n*=3) HMECs, Prolif and senescent (M0/HIS) WT (*n*=3) and *BRCA1*^mut/+^ (*n*=3) HDEs, as well as Prolif and senescent (M1) WT (*n*=3) and *BRCA1*^mut/+^(*n*=3) HMFs and HDFs. (**b**) ChIP analysis of SIRT1 abundance at telomeres in WT and *BRCA1*^mut/+^ HMECs. Data are presented as the average of three WT and three *BRCA1*^mut/+^ patient samples±s.e.m. (**c**) SIRT1 levels in shLuciferase (control) and shBRCA1 HMECs determined using western blot analysis. (**d**) SIRT1 levels (protein and mRNA) in shScr (control), shSIRT1-#1 and shSIRT1-#2 HMECs. Images of SA-β-galactosidase staining in shScr (control), shSIRT1-#1 and shSIRT1-#2 HMECs. (**e**, upper panel) Acetyl-pRb (Acetyl-Lysine blot) and total pRb levels in shScr (control), shSIRT1 and shBRCA1 HMECs determined with pRb IP and western blot. (lower panel) Western blot analysis of Acetyl-Lysine, total pRb and tubulin (loading control) levels in total cell lysates from shScr (control), shSIRT1 and shBRCA1 HMECs. (**f**) Western blot analysis of Acetyl-H3K9, total H3, Acetyl-H4K16 and total H4 levels in shScr (control), shSIRT1 and shBRCA1 HMECs from Patient 1 and Patient 2. (**g**) ChIP analysis of Acetyl-H4K16 and Acetyl-H3K9 abundance at telomeres in shScr (control), shSIRT1 and shBRCA1 HMECs from Patient 1 and Patient 2 (results were averaged). Student's two-tailed *t*-test was used to calculate *P* values. (*) indicates *P* value within the 0.05 level of significance. Error bar, s.e. Scale bar, 100 μm.

Examination of SIRT1 levels in HMECs from *BRCA1*-mutation carriers revealed significantly reduced protein expression in senescent cells (*t*-test *P*=0.019; Fig. 6a, Supplementary Fig. 7a,b). The decrease in SIRT1 was cell-type-specific as the levels of SIRT1 in senescent *BRCA1*^mut/+^ HDEs, HMFs or HDFs did not differ from those found in senescent WT cells of the same tissue origin (Fig. 6a, Supplementary Fig. 7a,b). SIRT1 occupancy was also examined at telomeres and its levels were found to be significantly reduced in *BRCA1*^mut/+^ compared with WT HMECs (*t*-test *P*=0.017; Fig. 6b). Consistent with the notion that *SIRT1* is a BRCA1 target^44^, SIRT1 levels were decreased in WT HMECs in which BRCA1 expression had been attenuated through lentiviral-mediated short hairpin inhibition (Fig. 6c). Furthermore, knockdown of SIRT1 in WT HMECs resulted in cell-cycle arrest and morphological changes associated with senescence (Fig. 6d). The decrease in SIRT1 expression was also associated with increased Ac-pRb (as well as increased acetylation of other proteins) in HMECs following knockdown of BRCA1 or SIRT1 (Fig. 6ei,ii). Histone substrates of SIRT1, specifically H4K16 acetylation, were also found to be altered in HMECs in which BRCA1 or SIRT1 was inhibited. Global as well as telomere-specific levels of Ac-H4K16 were markedly increased in shBRCA1 and shSIRT1 HMECs, while no significant changes in levels of Ac-H3K9 were observed (Fig. 6f,g). These findings imply that BRCA1 haploinsufficiency in HMECs, but not in other cell types examined, is associated with misregulation of SIRT1. Decrease in SIRT1 levels leads to accumulation of Ac-H4K16 and Ac-pRb, thereby resulting in telomere erosion, genomic instability and pRb-dependent premature senescence.

### Evidence of SIRT1 misregulation and HIS *in vivo*

**Figure 7:**
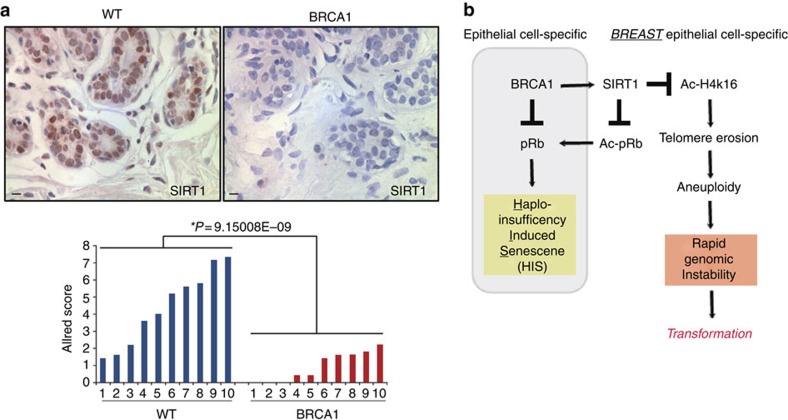
Evidence of SIRT1 misregulation *in vivo*. (**a**) Images of IHC staining and quantification of SIRT1 levels in epithelial cells from WT (*n*=10) and *BRCA1*^mut/+^ (*n*=10) breast tissues. Allred score methodology was used to measure SIRT1 antibody staining. Student's two-tailed *t*-test was used to calculate *P* value. (*) indicates *P* value within the 0.05 level of significance. (**b**) Model of tissue- and cell-type-specific response to BRCA1 haploinsufficiency. Scale bar, 10 μm.

To determine whether SIRT1 misregulation and HIS might be observed *in vivo*, we examined disease-free breast tissue specimens from *BRCA1*-mutation carriers for SIRT1 expression and evidence for pRb pathway activation. Semiquantitative immunohistochemistry (IHC) was applied to disease-free prophylactic mastectomy tissues obtained from *BRCA1*^mut/+^ carriers and age-matched reduction mammoplasty tissues from WT non-carriers. Consistent with *in vitro* results, SIRT1 expression and nuclear localization were significantly reduced in luminal cells within lobules of *BRCA1*^mut/*+*^ breast tissues compared with their WT counterparts (Fig. 7a, *t*-test *P*=9.15 × 10^−9^).

Gene expression data collected from freshly isolated breast epithelial cells from WT (*N*=4) and *BRCA1*-mutation carriers (*N*=4) was also queried to determine whether evidence of DDR and HIS pathway activation could be observed *in vivo*. Consistent with *in vitro* findings, Ingenuity Pathway Analysis revealed that in addition to double-strand break repair, and mismatch repair, ATM signalling (*t*-test *P*=5.83 × 10^−3^) was significantly enriched in *BRCA1*^mut/+^ tissues. Moreover, comprehensive network analysis using ingenuity gene network analysis revealed 25 significant networks as major regulators in epithelial cells from *BRCA1*-mutation carriers, 12 of which formed an overlapping network with central nodes consisting of SIRT1, cyclin D1, CDKN1A and p53 (Supplementary Fig. 8). Interestingly, additional networks involving cellular stress, metabolism and autophagy were also enriched *in vivo* in *BRCA1*^mut/+^ tissues, consistent with the role of these pathways in regulating autophagy and senescence in response to DNA damage and chronic apoptotic stress^45^,^46^,^47^,^48^. Collectively, these findings suggest that breast epithelial cells in tissues of *BRCA1*-mutation carriers also exhibit misregulation of SIRT1 expression as well as DDR and pRb pathway activation before the onset of cancer.

## Discussion

Over the past 20 years, tremendous progress has been made in understanding how *BRCA1* loss leads to defects in DDR, chromatin organization, gene transcription, protein stability and cell division^8^,^9^,^10^. Despite this information, it has still remained unclear why mutation in a single copy of *BRCA1* leads to rapid tumour onset in such a tissue- and cell-type-specific manner. In this study, we have provided evidence of a tissue- and cell-type-specific difference in BRCA1 function. Our data suggest that inherited mutations in *BRCA1*^mut/+^ HMECs but not other cell types lead to decreased SIRT1 expression, rapid telomere erosion and genomic instability (Fig. 7b). We found that the decrease in overall SIRT1 levels in *BRCA1*-haploinsuficient HMECs also results in reduced SIRT1 localization at telomeric regions of chromatin. When tested for the consequences of inhibiting BRCA1 or SIRT1 in HMECs, we found increased histone acetylation (H4K16) globally as well as at telomeres; the latter heterochromatin changes are linked with increase in telomere fragility^42^,^43^. Indeed, loss of telomeric heterochromatin causes telomere attrition and increased telomere dysfunction leading to rapid genomic instability^49^. Together, these data suggest that the regulation of SIRT1 by BRCA1 in a cell-type-specific manner affects telomere as well as genomic stability.

In this study we also found that *BRCA1*-haploinsufficient epithelial cells, but not fibroblasts, undergo a novel form of premature senescence associated with haploinsufficiency rather than loss of a tumour suppressor (Fig. 7b). Epithelial cells undergoing HIS displayed typical phenotypes of senescence such as: morphological changes, SA-β-galactosidase positivity, increased SASF expression and cell-cycle arrest mediated by the active pRb signalling pathway. Our data suggest that in HMECs, since INK4a/p16 expression is lost, active pRb signalling pathway is induced through increased levels of pRb acetylation, while in HDEs it is induced by increased INK4a/p16 expression. Surprisingly, given the well-established role of p53 in mediating growth arrest in response to *BRCA1* loss, HIS in epithelial cells did not correlate with the increased p53 signalling pathway. However, p53 involvement in HIS is not completely excluded in this study and further genetic validations are needed to support this conclusion. Furthermore, as the name of this senescence alludes, HIS was not associated with loss of the remaining WT *BRCA1* allele in either breast or skin epithelial cells. The finding that epithelial cells and not fibroblasts undergo HIS when harbouring a single mutant allele of *BRCA1* highlights the important but not well-established notion that the molecular circuitry of senescence responses in fibroblasts differ from those in epithelial cells.

Senescence is not a full-proof mechanism to prevent neoplastic transformation, as it has been shown to be readily bypassed following p53 and pRb loss^50^. Because p53 is frequently mutated or lost in *BRCA1*-associated breast cancers^20^,^51^, it is thought to be the major pathway in suppressing cellular proliferation during *BRCA1*-associated cancer progression. However, we found an unexpected role for pRb in suppression of cellular proliferation in *BRCA1*-haploinsufficient cells. Indeed, the importance of overcoming this pRb-mediated proliferative barrier during cancer progression is supported by the high incidence of *RB1* loss or mutations in human breast cancers with inactivated *BRCA1* (refs 51, 52, 53). The finding that *BRCA1*^mut/+^ HMECs exhibit even greater genomic instability and telomeric fusions following forced proliferation beyond HIS *in vitro* suggest that the proliferative barriers imposed by p53 and pRb likely impose a strong selective pressure that must be overcome before *BRCA1* loss during neoplastic transformation *in vivo*. This is consistent with recent findings in which immortalized mammary epithelial cells genetically engineered to harbour a single-allelic *BRCA1* mutation were unable to survive on loss of the WT allele while in the presence of intact cell-cycle checkpoints^20^. Moreover, mutations in PTEN and p53 have been reported to precede *BRCA1* LOH in *BRCA1*-associated breast tumours^15^. Thus, our results combined with those of others support the notion that *BRCA1* LOH is not an obligatory early step in *BRCA1*-associated tumour progression and that *BRCA1* LOH is likely to occur following the abrogation of other tumour-suppressive networks. Rather, the idea of ‘obligatory haploinsufficiency'^30^ is better suited to describe *BRCA1*-associated tumour progression, since decreased levels of BRCA1 are sufficient to produce abnormal cellular phenotypes. Consistent with this, partial loss of BRCA1 function leads to rapid telomere dysfunction and genomic instability, suggesting that mutation in a single copy of *BRCA1* is sufficient to induce a mutator phenotype driven by genetic and epigenetic events activating a novel form of senescence. Since *BRCA1* haploinsufficiency imposes such a strong selective pressure to mutate or lose p53 and pRb pathways, this likely sets the stage for accelerated evolution and cancer formation in a tissue-specific manner and may provide an explanation for the rapid and early-onset pattern of tumour formation in *BRCA1*-mutation carriers. Furthermore, while breast and ovarian cancers are the most widely discussed tissues that *BRCA1*-mutation carriers are predisposed to, it has been reported that the odds ratio for developing basal cell carcinoma in *BRCA1*-mutation carriers is significantly higher than in non-mutation carries^54^. Therefore, the observation that keratinoctyes from *BRCA1*-mutation carriers undergo premature senescence further supports the hypothesis that this type of premature senescence might be at the heart of the tissue-specific oncogenic properties of the loss of BRCA1. These ideas would greatly benefit from future studies demonstrating HIS in a variety of epithelial tissues (ovarian epithelial cells as well as non-breast, non-ovarian epithelial cells common in cancer) that are either *BRCA1*^mut/+^ or generated with a modest activity shBRCA1 that would mimic the hemizygous state.

Our findings combined with those of others demonstrating that haploinsufficiency rather than nullizyogisity for *BRCA1* leads to rapid genomic instability in breast epithelial cells^15^,^20^,^21^ seems to conflict with observations in rodents. Although several mouse models of BRCA1 deficiency exist, they have been unable to recapitulate many of the features of *BRCA1* mutation in humans, including defects in mammary differentiation or increased frequency of tumour formation^4^,^5^,^16^,^17^. In fact, *BRCA1* heterozygous mice do not exhibit any apparent phenotype nor do they develop spontaneous mammary tumours^1^,^7^. Furthermore, conditional deletion of *BRCA1* in mouse mammary epithelial cells does not result in accelerated tumour formation or increased genomic instability; rather, these mice develop mammary tumours at a low frequency and late in life and only on the background of additional genetic mutations such as heterozygosity for *p53* (refs 55, 56). This difference could be because the molecular and genetic requirements for cellular transformation are not well modelled in rodents. This is particularly relevant for pRB, BRCA1 and telomere biology where there are significant differences between mice and humans^57^,^58^,^59^. Since these are the major pathways implicated in premalignant features associated with *BRCA1* haploinsufficiency in human cells, modelling *BRCA1*-associated phenotypes in mice might require further affecting telomere stability or pRb. Future studies evaluating whether HIS is observed in the context of other tumour-suppressor genes and whether it is associated with cell-type-specific predisposition to cancer are warranted. This will help broaden the knowledge about tumour-suppressor genes beyond their generalized division into the ‘gatekeepers' and ‘caretakers' as well as improve our understanding of the requirements for neoplastic transformation in a tissue- and cell-type-specific manner.

## Methods

### Cell lines and tissue culture

All human breast tissue procurement for these experiments was obtained in compliance with the laws and institutional guidelines, as approved by the Institutional Review Board committees from Brigham and Women's Hospital and Tufts Medical Center. Disease-free prophylactic mastectomy and skin tissue derived from women carrying a known deleterious *BRCA1* heterozygous mutation were obtained with patient consent from the Surgical Pathology files or immediately following prophylactic mastectomy surgery. Tissues in which *BRCA1* mutation was confirmed but not known were submitted for sequence/genotyping at Myriad Genetic Laboratories to confirm *BRCA1* mutation. *BRCA1*-mutation status is listed in Supplementary Table 1. The range of patient ages for fresh *BRCA1*^mut/+^ tissue used in this study was 35–53 with a median age of 44. *BRCA1*^+/+^ tissues were obtained from discarded material at Tufts Medical Center undergoing elective reduction mammoplasty at Tufts Medical Center. All disease-free breast tissues were verified by surgical pathologists before use in these studies. The range of patient ages for fresh *BRCA1*^+/+^ tissue used in this study was 30–54 with a median age of 40.

HMECs were isolated from breast tissues that were minced and enzymatically digested overnight with a mixture of Collagenase and Hyluronidase^17^. Digested cells were plated briefly in serum (1–2 h) to deplete mammary fibroblasts from the organoid fraction (epithelium). The organoids were dissociated to single-cell suspension by trypsinization and were filtered through a 40-μm mesh (BD Biosciences) to remove clumps. Immediately after dissociation, cells were plated and from then on cultured in MEGM (Lonza) supplemented with bovine pituitary extract, insulin (5 μg ml^−1^), EGF (10 ng ml^−1^) and hydrocortisone (1 μg ml^−1^). These cells were immortalized with the catalytic subunit of human telomerase (hTERT) after stasis^60^. HMF were obtained from the single-cell fraction of digested breast tissue after overnight incubation with Collagenase and Hyluronidase^17^ and were subsequently cultured in DMEM (Invitrogen) supplemented with 10% Calf Serum.

In order to isolate keratinocytes (HDEs) and HDF, skin tissue was chopped up into 0.5-cm^2^ cubes using a razor blade and were incubated overnight for digestion in a Dispase-containing solution^61^. The following day, epidermis and dermis layers were separated and incubated in Collagenase-containing solution for 20 min at 37 °C. Tissue/cell suspensions were pelleted, resuspended in trypsin and frequently agitated to promote the dissociation of cells^61^. The dissociated epidermis layer was pelleted, plated and cultured in KGM-2 (Lonza) supplemented with bovine pituitary extract, insulin (5 μg ml^−1^), human Epidermal Growth Factor (hEGF) (10 ng ml^−1^), hydrocortisone (1 μg ml^−1^), GA-1,000 (gentamicin, amphotericin-B), Epinephrine and Transferrin. This dissociated dermis layer was pelleted, plated and cultured in DMEM (Invitrogen) supplemented with 10% Calf Serum.

### Lentiviral constructs and virus production

The VSV-G-pseudotyped lentiviral vectors were generated by transient co-transfection of the vector construct with the VSV-G-expressing construct pCMV-VSVG^62^ and the packaging construct pCMV DR8.2Dvpr^62^, generously provided by Inder Verma, into 293T cells together with FuGENE 6 transfection reagent (Roche). Lentiviral shRNA constructs targeting BRCA1, SIRT1 and pRb (Sigma-Aldrich, MISSION shRNA SHCLNG-NM_007294, SHCLNG-NM_012238 and SHCLNG-NM_000321, respectively) were prepared according to the manufacturer's protocol. All shRNA sequences used in this study are provided in Supplementary Table 3.

### Western blot analysis

Cultured cells were harvested by trypsinization, pelleted and incubated in RIPA buffer supplemented with protease and phosphatase inhibitors (Roche) to obtain whole-cell lysates. Cellular debris was removed by centrifugation at 18,000*g* for 10 min. Overall, 30 μg of the whole-cell lysate was used per sample. Western blot analysis was performed according to the manufacturer's protocol (Bio-Rad). Briefly, 12% and/or 4-12% pre-cast gels (depending on the kDa size of the proteins) and XT-MOPS running buffer were used for SDS–PAGE electrophoresis. Nitrocellulose membrane (0.2 or 0.45 μm) was used for protein transfer. Membranes were incubated overnight at 4 °C with primary antibodies diluted in 1% bovine serum albumin in TBS-T. Secondary antibodies were applied for 1 h at room temperature. The antibodies used include the following: BRCA1 (Cell Signaling #9010, 1:1,000), p16 (Santa Cruz sc-377412, 1:500), p53-Ser15 (Cells Signaling #9284, 1:1,000), p53-total (Santa Cruz sc-126, 1:500), p21 (Santa Cruz sc-397, 1:500), γH2AX (Cell Signaling #9718, 1:1,000), p27 (Santa Cruz sc-528, 1:500), pRb-Ser795 (Cell Signaling #9301, 1:1,000), pRb-total (Santa Cruz sc-50, 1:2,000), Cyclin E (Santa Cruz sc-481, 1:500), Cyclin A (Santa Cruz sc-751, 1:500), SIRT1 (Millipore 04-1557, 1:1,000) and β-actin (AbCam ab6276, 1:5,000). Uncropped versions of most relevant western blots are provided in Supplementary Fig. 9.

### Immunoprecipitation

shRNA-expressing WT HMECs (shScr, shBRCA1 and shSIRT1) were lysed in IP buffer (20 mM Tris pH 7.5, 150 mM NaCl, 1 mM EDTA, 1 mM EGTA and 1% Triton X-100) supplemented with protease and phosphatase inhibitors (Roche). For immunoprecipitation assays, protein lysates (200–600 μg) were combined with 2 μg of antibody and 25 μl of Protein A/G Plus agarose beads (Santa Cruz, sc-2003). Following an overnight incubation at 4 °C, agarose beads were extensively washed in IP buffer, resuspended in SDS sample buffer (125 Mm Tris pH 6.8, 2.5% SDS, 10% glycerol, 2.5% 2-mercaptoethanol, 0.01% bromophenol blue) and loaded into a protein gel. Antibodies used in these experiments included anti-pRB (BD Pharmingen, #554136) and anti-Acetylated lysine (Cell Signaling, #9441).

### Histone acid extractions and blots

Cells were harvested by trypsinization and acid extraction of histone proteins was carried out by lysing cells in PBS with 0.5% Triton X-100, 2 mM phenylmethyl sulphonyl fluoride (PMSF) and 0.02% NaN_3_. Nuclei were pelleted by centrifugation at 1,000*g* and the nuclear pellet was incubated at 4 °C overnight in 0.2 N HCl 63. Western blot analysis was carried out as described above using 5 μg of acid-soluble lysate per sample. Antibodies used were as follows: anti-histone H3 (Cell Signaling #9715, 1:1,000), anti-acetyl-histone H3K9 (Cell Signaling #9649P, 1:1,000), anti-histone H4 (Millipore 07-108, 1:250) and anti-acetylated histone H4K16 (Millipore 07-108, 1:500).

### Senescence-associated β-galactosidase assay

Senescence-associated β-galactosidase staining was performed on cells cultured in six-well plates. The cells were initially fixed with formaldehyde/glutaraldehyde solution. After fixation, the cells were washed twice in PBS. Samples were covered with staining solution and incubated overnight (12–16 h) at 37 °C (no CO_2_)^64^. Images were captured by brightfield microscopy.

### Quantitative RT–PCR

Total RNA from cultured cells was extracted with the RNeasy Mini Kit (QIAGEN). cDNA was prepared with an iScript kit (Bio-Rad), and PCR was carried out with SYBR Green (Bio-Rad). A list of all primers used in this study is provided in Supplementary Table 3. Analysis was performed with the delta *C*_t_ method.

### Immunofluorescence

Cells were cultured on eight-well chamber slides and fixed with methanol at −20 °C for 10 min. Samples were incubated overnight at 4 °C with primary antibodies diluted in 1% bovine serum albumin PBS. Fluorescently labelled secondary antibodies were applied for 1 h at room temperature. Cells were counterstained with 4,6-diamidino-2-phenylindole (DAPI). A Nikon Eclipse 80t microscope and SPOT camera were used for analysing and photographing the stained sections. The antibodies used included the following: Ki-67 (AbCam ab15580, 1:200), γH2AX (Cell Signaling #9718, 1:100), phospho-p53BP (Cell Signaling, #2675, 1:100) and pATM/ATR substrate (Cell Signaling #2851, 1:100).

### Telomere chromatin immunoprecipitation and qPCR

In brief, after crosslinking and sonication^41^, chromatin from 4 × 10^6^ cells was aliquoted and incubated with protein A/G Plus agarose beads (Santa Cruz Biotechnology, sc-2003) and the following antibodies: 5 μg of anti-histone H3 (#ab1791, Abcam), 5 μg of anti-H3K9 (#H9286, Sigma), 5 μg anti-histone H4 (#ab10158, Abcam), 5 μg of anti-H4K16Ac (#39167, Active Motif) or pre-immune serum. The immunoprecipitated DNA was transferred to a Hybond N+ membrane using a dot blot apparatus. The membrane was then hybridized with a telomeric probe containing TTAGGG repeats. Quantification of the signal was performed with the ImageJ software. The amount of telomeric DNA after chromatin immunoprecipitation (ChIP) was normalized to the total telomeric DNA signal for each genotype (input), as well as to the H3 and H4 abundance at these domains, thus correcting for differences in the number of telomere repeats or in nucleosome spacing.

ChIPs on *BRCA1*^mut/+^ and WT HMECS were performed according to the following protocol: crosslinked nuclei were sonicated to 150–500 bp DNA fragments in buffer containing 1% SDS, 50 mM Tris-HCl (pH 8.0), 10 mM EDTA, 1 mM PMSF and complete protease inhibitors (Roche), and bound ChIP complexes were washed according to the Upstate/Millipore protocol^48^,^65^. Antibodies used were as follows: anti-SIRT1 (Cyclex Co, Ltd, Japan), anti-H4K16ac (Millipore, MA, USA) and anti-histone H3 (Abcam, UK). Quantitative PCR analysis of telomeric sequences was performed as described previously^12^, using forward primer (5′-CGGTTTGTTTGGGTTTGGGTTTGGGTTTGGGTTTGGGTT-3′) and reverse primer (5′-GGCTTGCCTTACCCTTACCCTTACCCTTACCCTTACCC-3′) at an annealing temperature of 60 °C.

### Immunohistochemistry

IHC was performed on formalin-fixed, paraffin-embedded tissue sections with sodium citrate antigen retrieval, followed by visualization with the ABC Elite peroxidase kit and DAB substrate (Vector Labs) for detection of SIRT1 (Millipore 04-1557, 1:100). IHC results were semiquantitatively analysed using the Allred Score^17^.

### Chromosomal metaphase analysis

Cultures were checked for harvest on the third day after trypsinization, and 30 μl of colcemid (10 μg ml^−1^ Gibco) was added per 5 ml of culture medium. Cultures were incubated for 30 min at 37 ^o^C. Cells were detached from flasks with trypsin and the supernatant and cells were spun at 1,100 r.p.m. for 5 min. The supernatant was discarded and replaced with 2:1 hypotonic solution (2 parts 0.075 M potassium chloride to one part 0.6% sodium citrate). The cultures were incubated at 37 ^o^C for 20 min and then fixed with several changes of fixative (methanol, acetic acid). Slides were prepared, treated with trypsin and stained with Wright's-Giemsa.

### Telomere length assays

The overall telomere lengths for each experimental sample were determined relative to the reference DNA by comparing the difference in their ratios of the telomere copy number (*T*) to the single copy gene copy number (*S*) using quantitative PCR. This ratio is proportional to the mean telomere length^66^. We used a modified qPCR assay for telomere sequence quantitation that is compatible with Applied Biosystems 7,900 HT instrumentation. Each plate (384 wells on each plate) contained a set of standards spanning an 81-fold range prepared by serial dilution, and each sample was analysed in triplicate. Two master mixes of PCR reagents were prepared, one with the telomere primers (telc and telg) and the other with either the albumin pair (albd and albu) or the beta-globin pair (hgbu and hgbd). A list of all primers is provided in Supplementary Table 3. The final concentrations in each PCR reaction were 0.8 × SYBR Green I Master Mix (Agilent Technologies), and 900 nM of the telomere pair, or 900 nM of the albumin pair, or 500 nM of the beta-globin pair. The thermal cycling profile used was 15 min at 95 °C, two cycles of 15 s at 94 °C, 15 s at 49 °C, followed by 32 cycles of 15 s at 94 °C, 10 s at 62 °C and 15 s at 74 °C with data acquisition. The plates were read at 74 °C to minimize the interference from the telomere primer dimers. The ABI software SDS version 2.0 was used to generate two standard curves from each plate, one for the telomere amplification, and the other for the single copy gene. The ratio (*T*/*S*) of the telomere copy number (*T*) to the single gene copy number (*S*) was generated for each experimental sample, and the value averaged across the triplicates, which provides the average telomere length for each experimental sample. The *T*/*S* ratios relative to the reference sample were generated using the comparative CT (cycle threshold) method^66^.

### Allele-specific LOH

PCR primers were designed flanking the *BRCA1* mutations from the individuals in the study (187delAG, 2800delAA, 5385insC, 4184del4, 4154delA and 943ins10). A list of all primers used is provided in Supplementary Table 3. PCR products were treated with ExoSap-It (USB) and sequenced. Sequence traces in the forward and reverse directions were compared between control blood DNAs of individuals with these germline mutations and the different derivatives of primary HMECs from individuals with these mutations using DNAstar 3.0 (www.dnastar.com). Loss was determined visually by two reviewers and it consisted of at least 30% difference between the two alleles compared with normal carrier ratios as described^67^.

### Quantitative telomere fluorescence *in situ* hybridization

For qFISH analysis on breast tissue samples, deparaffinated sections were hybridized with a PNA-tel Cy3-labelled probe^68^,^69^. DAPI and Cy3 signals were acquired simultaneously into separate channels using a confocal ultraspectral microscope Leica TCS-SP5, and maximum projections from image stacks were generated for image quantification.

For image acquisition we used a new tool for intelligent screening named ‘matrix screening remote control (MSRC)' developed at CNIO. The MSRC application manages a first fast scan with low-resolution settings, generating one image per sample of the whole tissue and later localizes the areas of interest, extracting their coordinates and surface area. With the spatial information, the MSRC application interacts with the microscope and load high-resolution settings, scanning automatically just the areas of interest.

Quantitative image analysis of telomere fluorescence intensity was performed on confocal images using the Definiens Developer Cell software (Definiens Developer XD). The DAPI image was used to define the nuclear areas that were separated by a Cellenger Solution. After defining the nuclear areas a predefined Ruleset was used for the quantification of telomere fluorescence intensity (Cy3 image). The fluorescence values for each section were exported to GraphPad Prism, and graphs were generated. The total number of telomeric spots scored for each genotype is shown. Student's *t*-test was used for statistical analysis.

### GSEA and IPA

GSEA was applied to previously published gene expression data collected on cultured proliferating primary HMECs isolated from *BRCA1*-mutation carriers (*N*=6) or age-matched WT (*N*=6; GSE19383, 23). Two-sided *t-*tests were run on the gene sets and the top 2,000 genes from each set were ranked. GSEA was performed as described previously^70^. Gene networks were constructed from our previously published gene expression data collected on freshly isolated HMECs isolated from *BRCA1*-mutation carriers (*N*=4) or age-matched WT (*N*=4; GSE25835, 17). Important hubs were identified using Ingenuity Pathway Analysis (Ingenuity Systems, Mountain View, CA) on the basis of differentially expressed genes between *BRCA1*^mut/+^ and WT patients (*n*=701 genes).

## Additional information

**How to cite this article:** Sedic, M. *et al.* Haploinsufficiency for BRCA1 leads to cell-type-specific genomic instability and premature senescence. *Nat. Commun.* 6:7505 doi: 10.1038/ncomms8505 (2015).

## Supplementary Material

Supplementary Information Supplementary Figures 1-9 and Supplementary Tables 1-3
