# Supplementary material for: Haploinsufficiency for BRCA1 leads to cell-type-specific genomic instability and premature senescence
Source: Nat Commun. 2015 Jun 24;6:7505. doi: 10.1038/ncomms8505 (PMC4491827; doi:10.1038/ncomms8505)
Supplement: Supplementary Information — Supplementary Figures 1-9 and Supplementary Tables 1-3 [file ncomms8505-s1.pdf]

Supplementary Figure 1

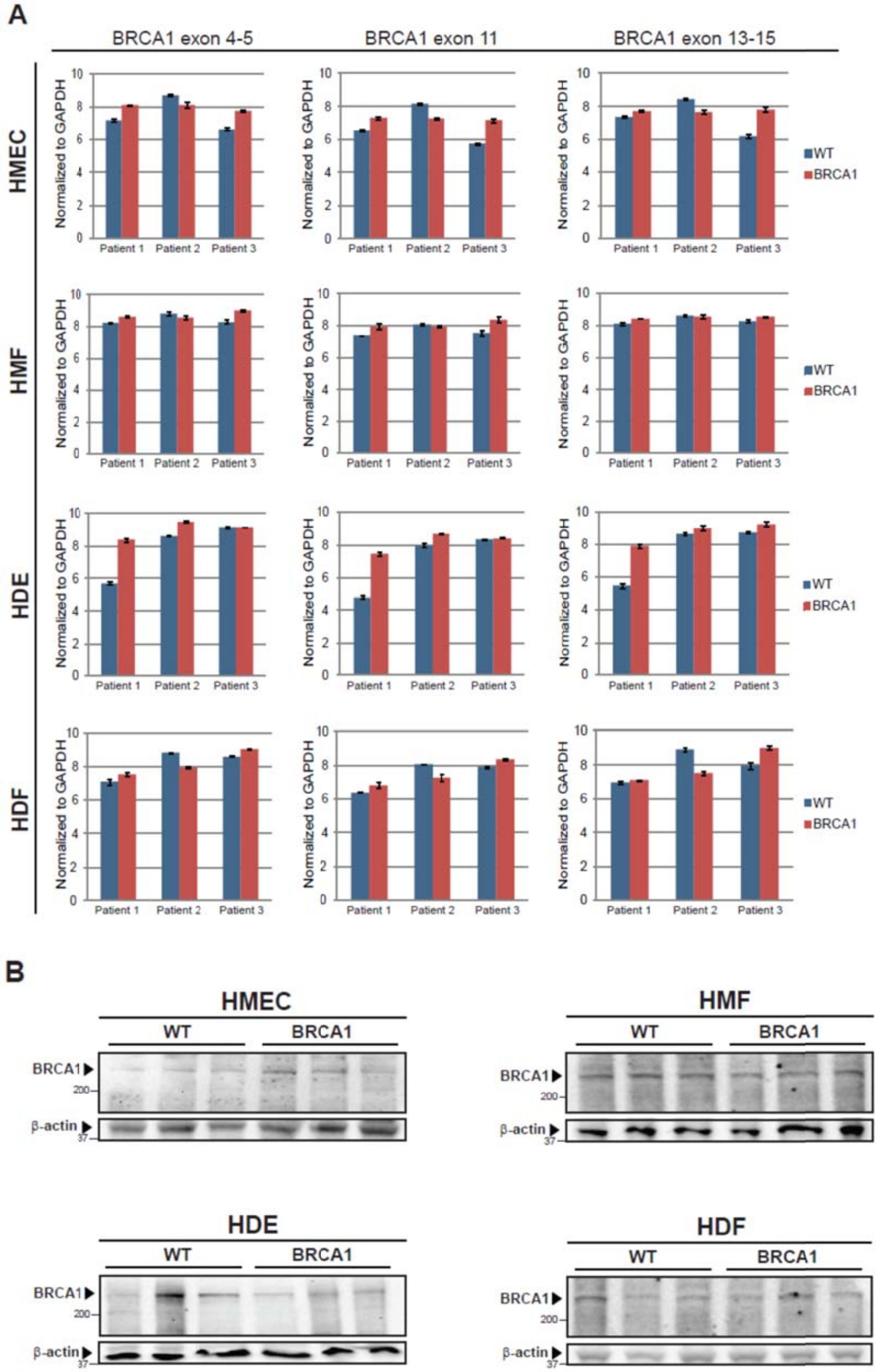

Supplementary Figure 1: BRCA1 levels in HMECs, HDEs, HMFs, and HDF. A) Analysis of full length BRCA1 mRNA transcript levels (exon 4-5, exon 11, exon 13-15) in WT and BRCA1 HMECs (n=3), HMFs (n=3), HDEs (n=3) and HDFs (n=3). The values were determined by qRT-PCR and normalized to GAPDH. B) BRCA1 protein levels in WT and BRCA1 HMECs (n=3), HMFs (n=3), HDEs (n=3) and HDFs (n=3) determined by western blot. Error bar = SE.

Supplementary Figure 2

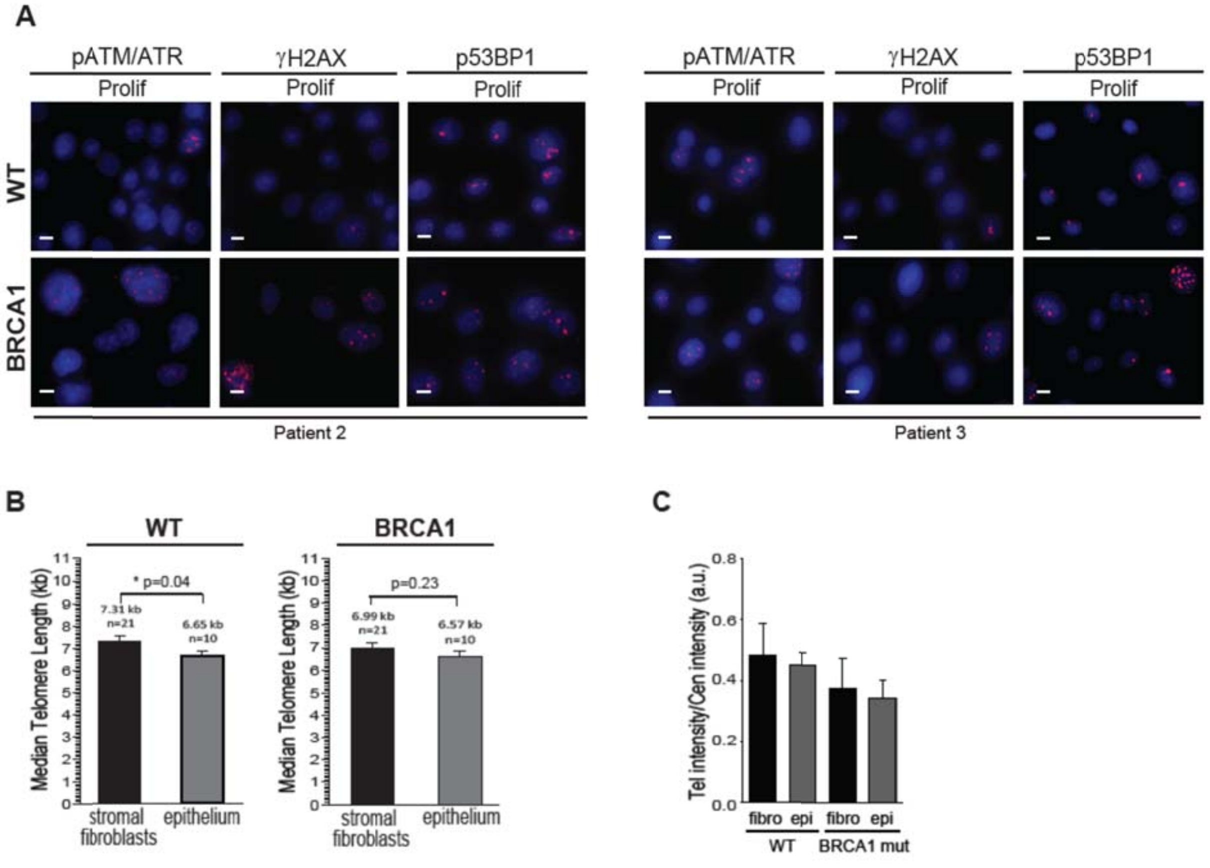

Supplementary Figure 2: BRCA1mut/+ HMECs exhibit increased DDR and telomere dysfunction. A) Representative images of immunofluorescence (IF) staining for phospho-ATM/ATR substrates, γH2AX foci, p53BP1 foci in proliferating (Prolif) WT and BRCA1mut/+ HMECs from additional patient samples. B) Median telomere length (kb) determined by qFISH of all epithelial cells (luminal and myoepithelial in both ducts and lobules) as well as in stromal fibroblast from in WT (n=21) and BRCA1mut/+ (n=10) disease-free patient tissues. C) qFISH for a centromeric probe to control for any putative probe accessibility problem. Student's two-tailed t test was used to calculate p values. Error bar = SE. Scale bar = 10µm.

Supplementary Figure 3

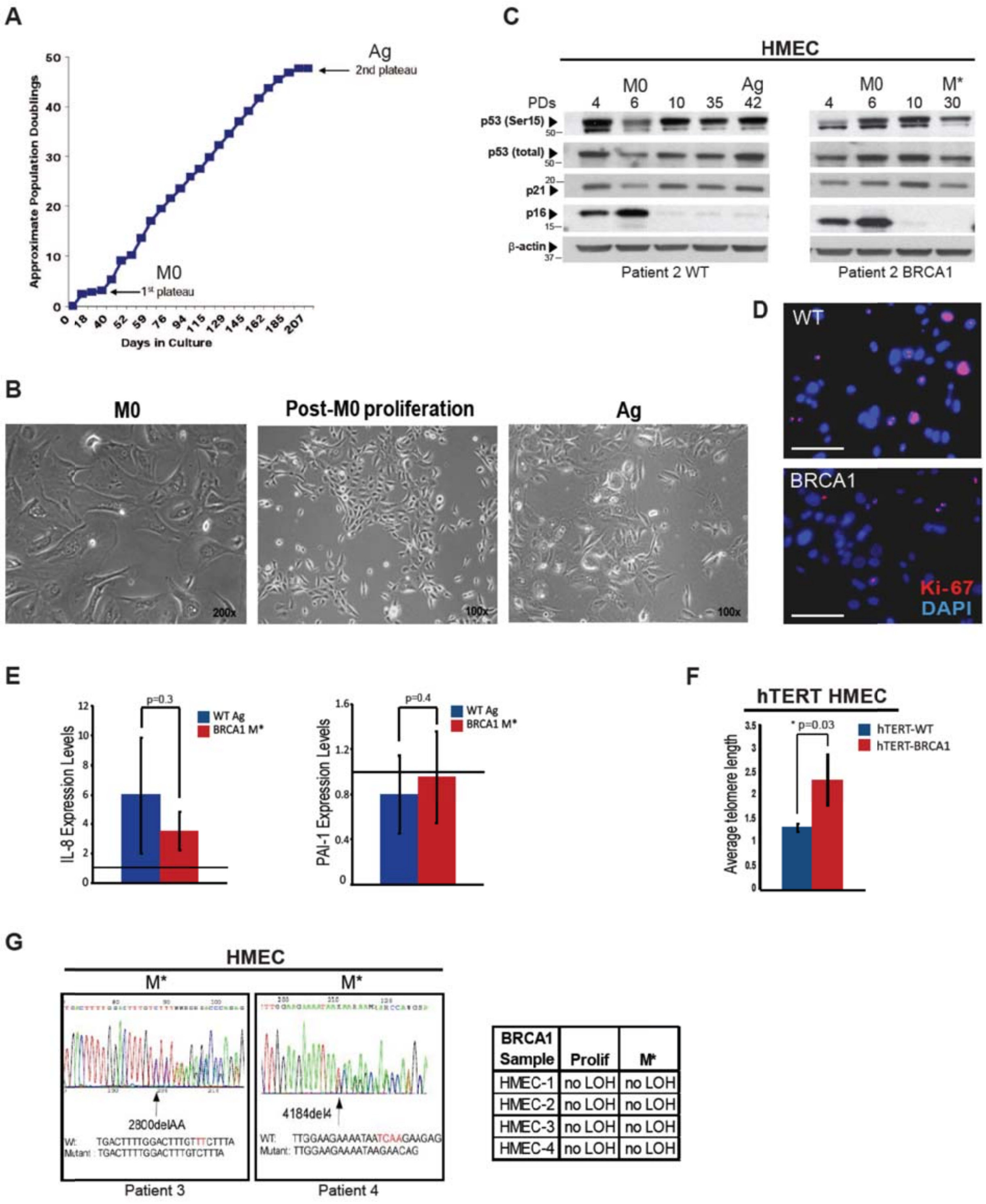

BRCA1 Sample	Prolif	M*
HMEC-1	no LOH	no LOH
HMEC-2	no LOH	no LOH
HMEC-3	no LOH	no LOH
HMEC-4	no LOH	no LOH

Supplementary Figure 3: BRCA1mut/+ HMECs undergo premature senescence. A) Representative growth curve of WT HMECs. M0 = Stasis, Ag = Agonescence B) Representative

phase-contrast brightfield images of WT HMECs in M0, post-M0 proliferation, and Ag. C) Western blot analysis of p16INK4a, total p53, p53 (Ser15), and p21 levels in WT and BRCA1mut/+ HMECs at indicated population doublings (PDs). D) Images of Ki67 positive cells determined by IF staining in Ag WT and M* BRCA1mut/+ HMECs. E) mRNA levels of IL-8 and PAI-1 (SASFs) in Ag WT and M* BRCA1mut/+ HMECs. The values were determined by qRT-PCR and normalized to proliferating cells (represented by line set at 1). F) Average telomere length in hTERT immortalized WT (n=3) and BRCA1mut/+ (n=3) HMECs. G) LOH analysis in M* BRCA1mut/+ HMECs from additional patient samples. Table summarizes data from LOH analysis of proliferating and M* BRCA1mut/+ HMECs. Student's two-tailed t test was used to calculate p values. Error bar = SE. Scale bar = 100µm.

Supplementary Figure 4

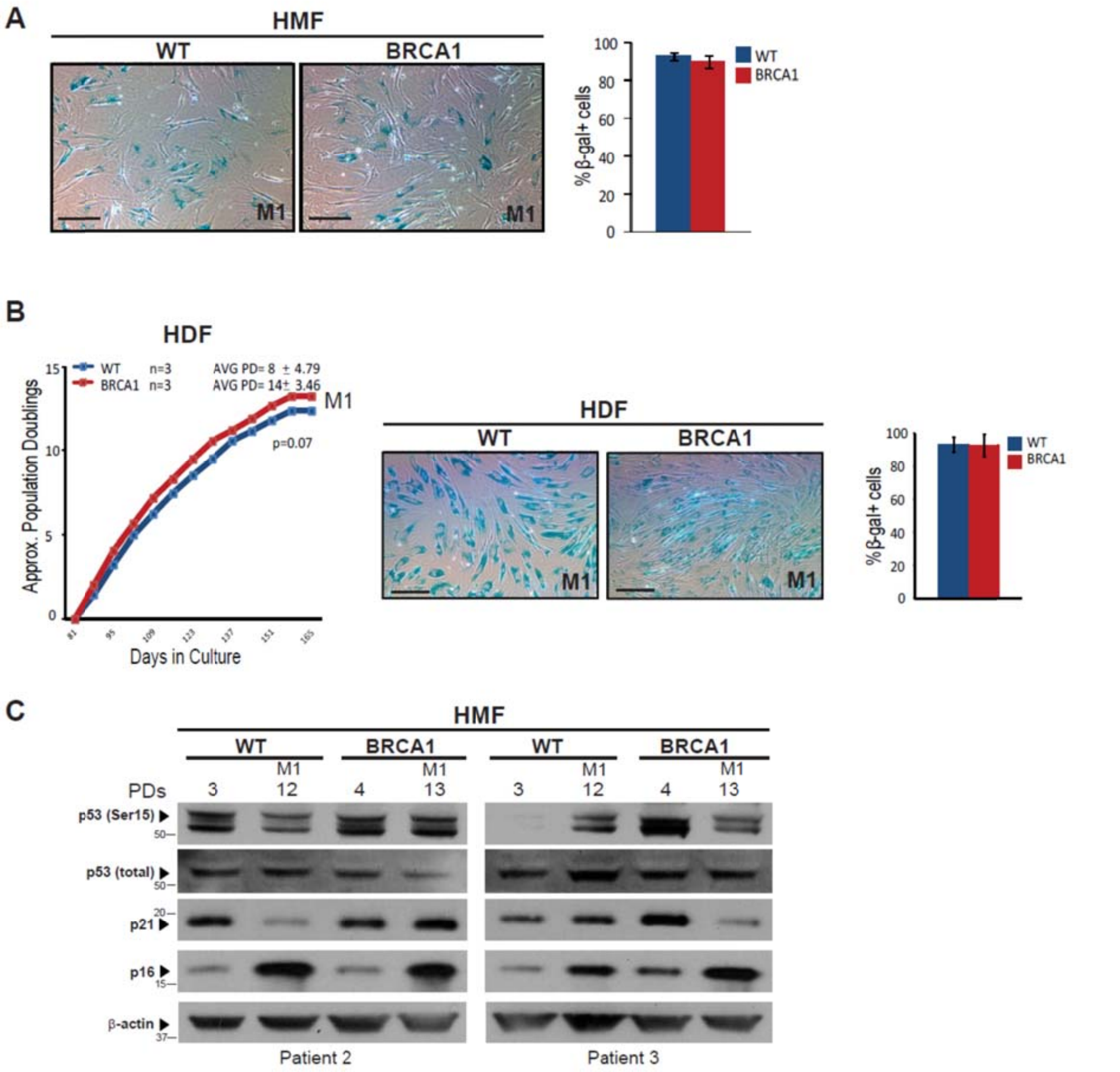

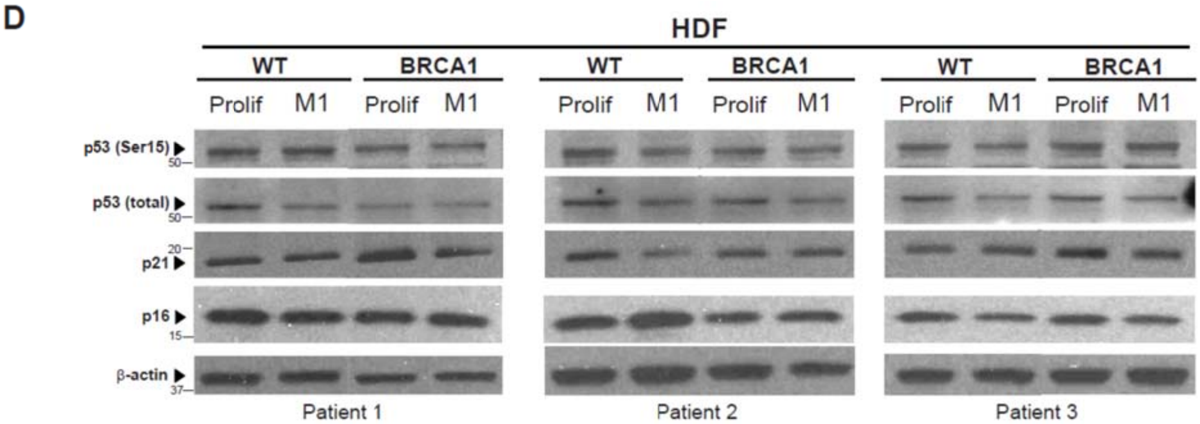

Supplementary Figure 4: Premature senescence is cell type specific. A) Brightfield images of SA-β-galactosidase staining and quantification of positive cells in M1 WT and BRCA1mut/+ HMFs. B) Representative growth curves of WT (n=3) and BRCA1mut/+(n=3) HDFs. Images and quantification of SA-β-galactosidase positive cells using β-galactosidase detection assay in senescent (M1) WT and BRCA1mut/+ HDFs. C) Western blot analysis of p16INK4a, total p53, p53 (Ser15), and p21 levels at indicated PDs in WT and BRCA1mut/+ HMFs from additional patient samples. D) Western blot analysis of p16INK4a, total p53, p53 (Ser15), and p21 levels in proliferating and M1 WT (n=3) and BRCA1mut/+ (n=3) HDFs. Student's two-tailed t test was used to calculate p values. Error bar = SE. Scale bar = 100µm.

Supplementary Figure 5

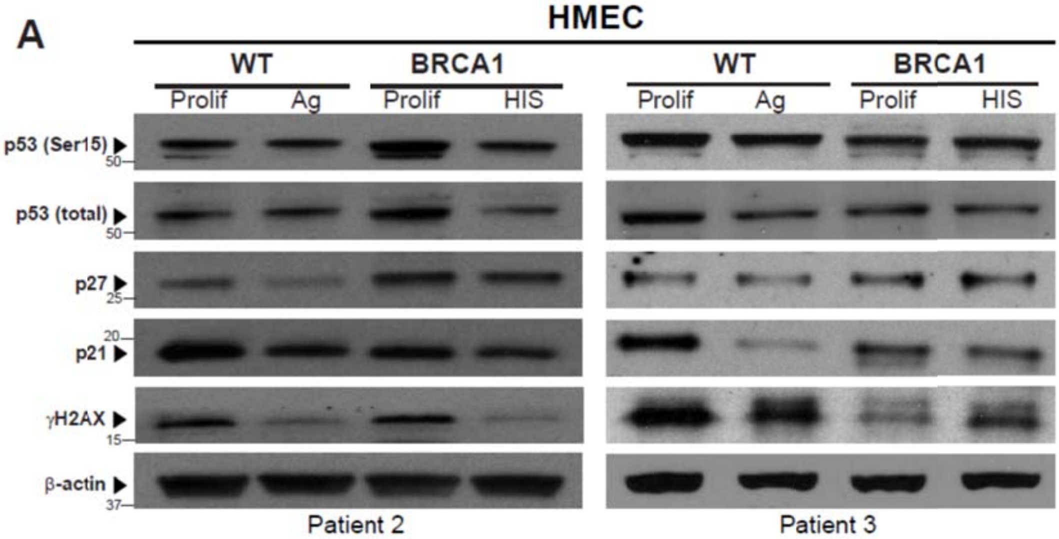

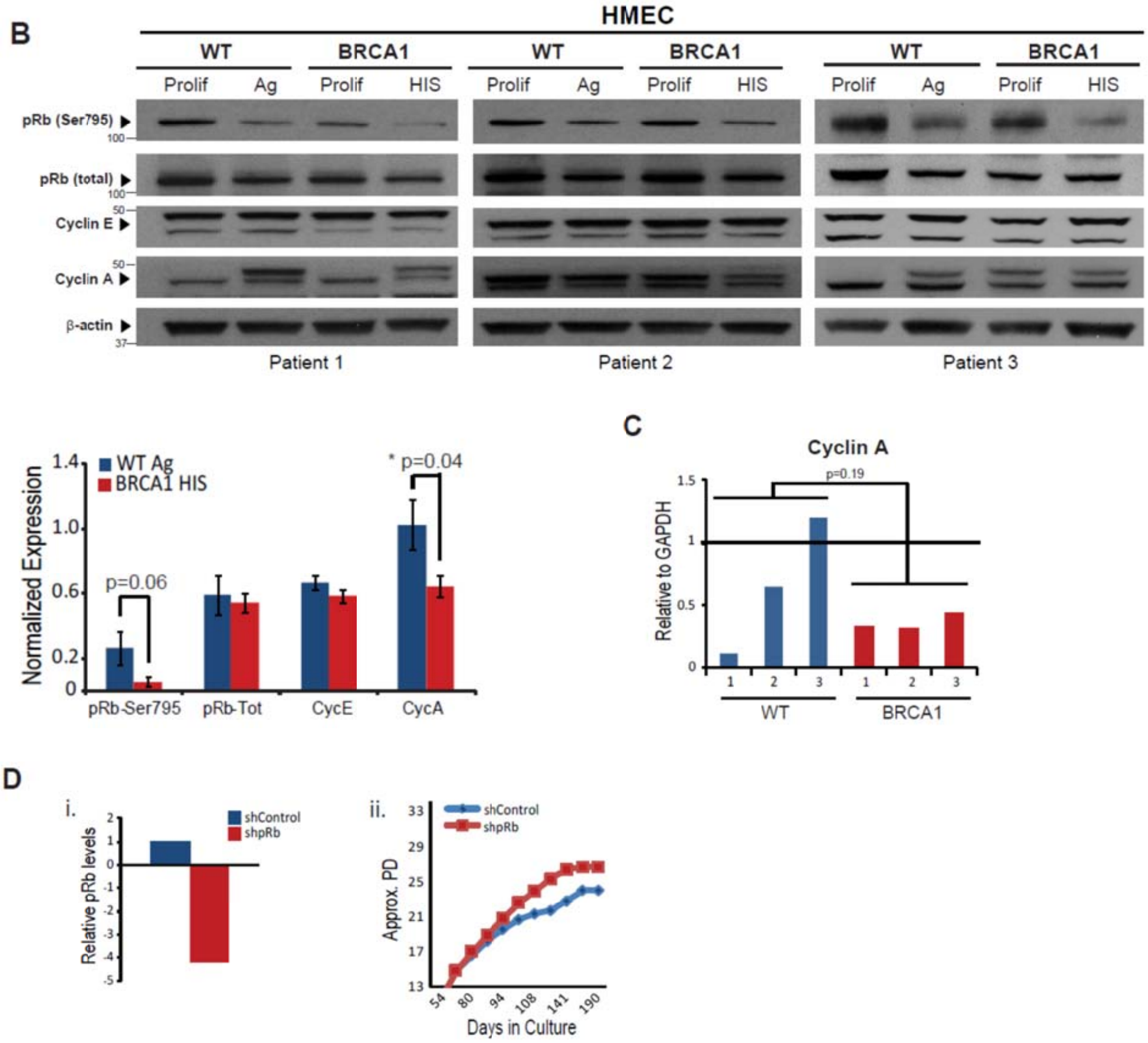

Supplementary Figure 5: p53 and pRb signaling pathway analysis in additional HMECs. A) Western blot analysis of p53 (Ser15), total p53, γH2AX, p21 and p27 levels in proliferating (Prolif) and agonescent/HIS (Ag/HIS) WT and BRCA1mut/+ HMECs from additional patient samples. B) Western blot analysis and quantification of phospho-pRb (Ser795), total pRb, Cyclin E and Cyclin A levels in proliferating (Prolif) and Ag/HIS WT and BRCA1mut/+ HMECs from additional patient samples. C) mRNA levels of Cyclin A in Ag WT and HIS BRCA1mut/+ HMECs. The values were determined by qRT-PCR and normalized to proliferating cells (represented by line set at 1). D) i. pRb knockdown in proliferating BRCA1mut/+ HMECs from Patient 2 (mRNA levels). ii. Growth curve of shScr (control) and shpRb BRCA1mut/+ HMECs from Patient 2. Student's two-tailed t test was used to calculate p values. Error bar = SE.

Supplementary Figure 6

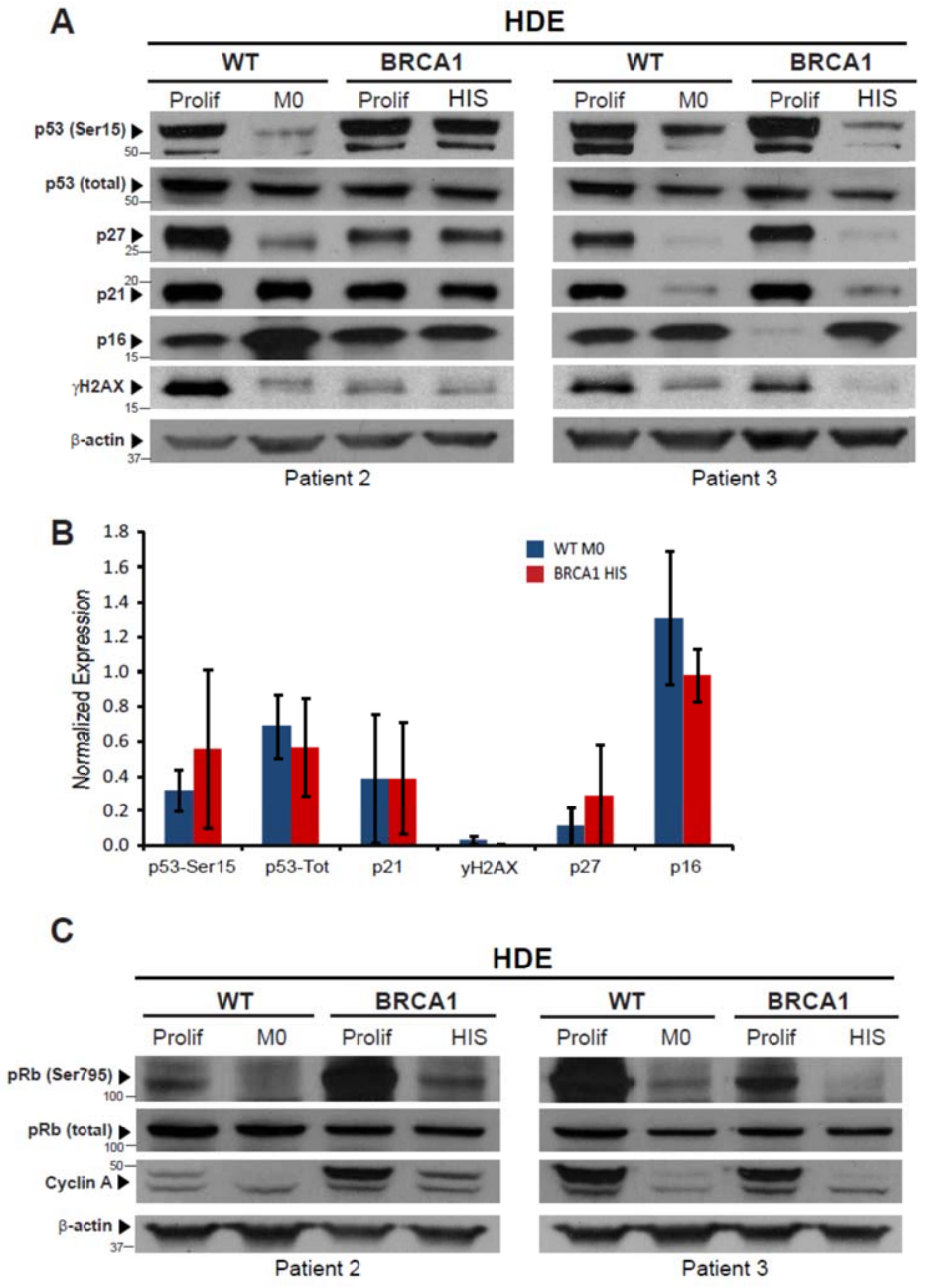

Supplementary Figure 6: p53 and pRb signaling pathway analysis in additional HDEs. A) Western blot analysis of p53 (Ser15), total p53, γH2AX, p21, p27 and p16INK4a levels in proliferating (Prolif) and growth-arrested (M0/HIS) WT and BRCA1mut/+ HDEs from additional patient samples. B) Quantification of p53 (Ser15), total p53, γH2AX, p21, p27 and p16 levels determined by western blotting in M0 WT (N=3) and HIS in BRCA1mut/+ (N=3) HDEs. C) Western blot analysis of phospho-pRb (Ser795), total pRb, and Cyclin A levels in

proliferating (Prolif) and M0/HIS WT and BRCA1mut/+ HDEs from additional patient samples. Error bar = SE.

Supplementary Figure 7

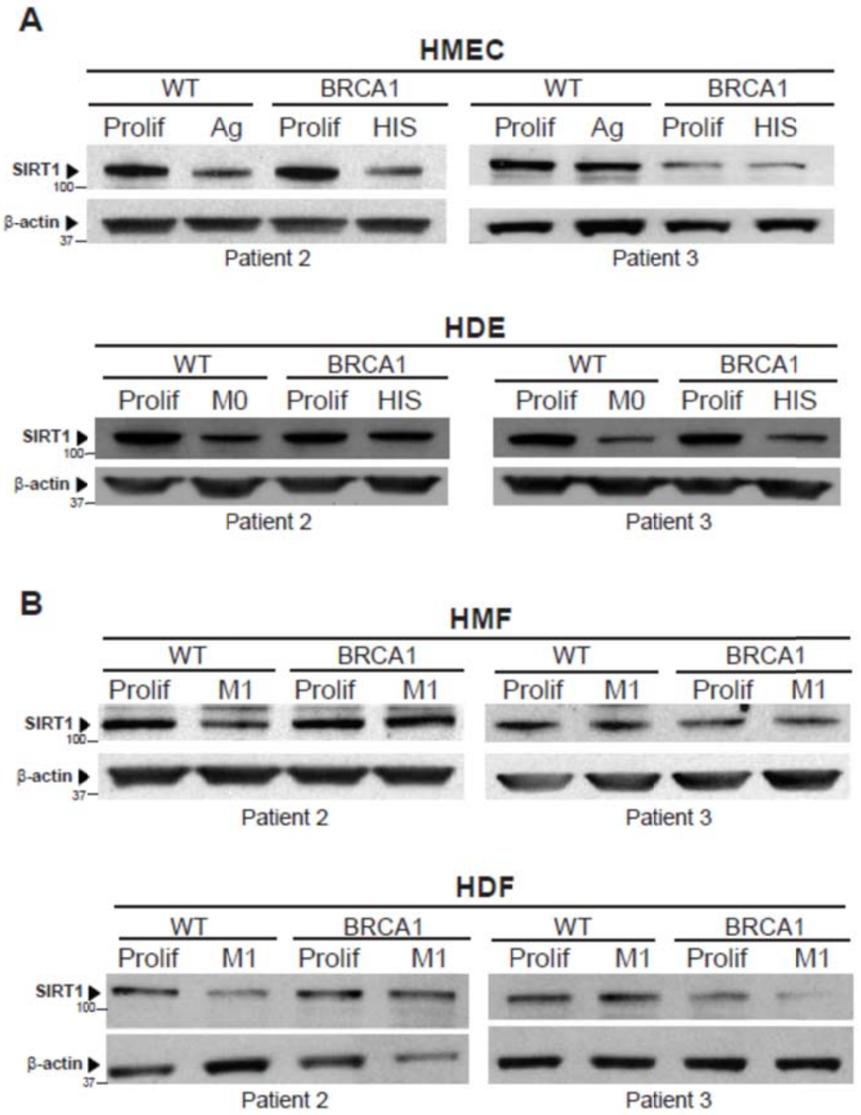

Supplementary Figure 7: **SIRT1 levels in additional patient samples.** A) Western blot analysis of SIRT1 levels in proliferating (Prolif) and Ag/HIS WT and *BRCA1*$^{mut/+}$ HMECs from additional patient samples. Western blot analysis of SIRT1 levels in proliferating (Prolif) and M0/HIS WT and *BRCA1*$^{mut/+}$ HDEs from additional patient samples. B) Western blot analysis of SIRT1 levels in proliferating (Prolif) and M1 WT and *BRCA1*$^{mut/+}$ HMF and HDFs from additional patient samples.

Supplementary Figure 8

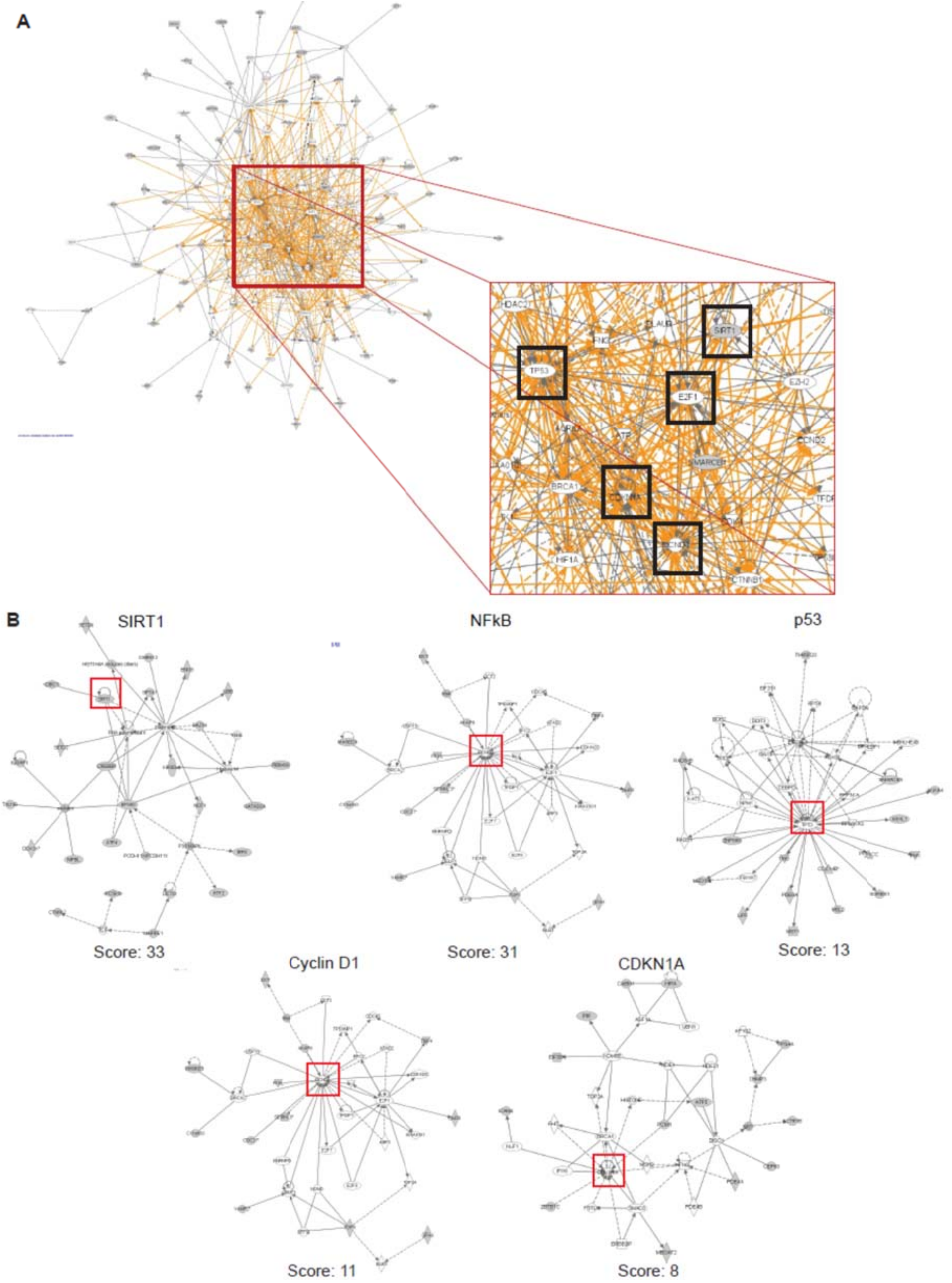

Supplementary Figure 8: Ingenuity Pathways Analysis Summary. A) Ingenuity Pathway Analysis identified 25 significant gene networks from differentially expressed genes from freshly isolated HMECs, as previously described (Proia et al. 2011, and as described in Materials and Methods). A gene network of 12 overlapping central nodes was constructed from this analysis. Genes colored in grey represent genes differentially expressed in BRCA1mut/+ tissues vs WT tissues. Nodes are displayed using various shapes that represent the functional class of the gene product. Edges with dashed lines show indirect interaction, while a continuous line represents direct interactions. B) The most significant gene networks identified involved central nodes in SIRT1, Rb, p53, and NFkappaB pathways.

Supplementary Figure 9

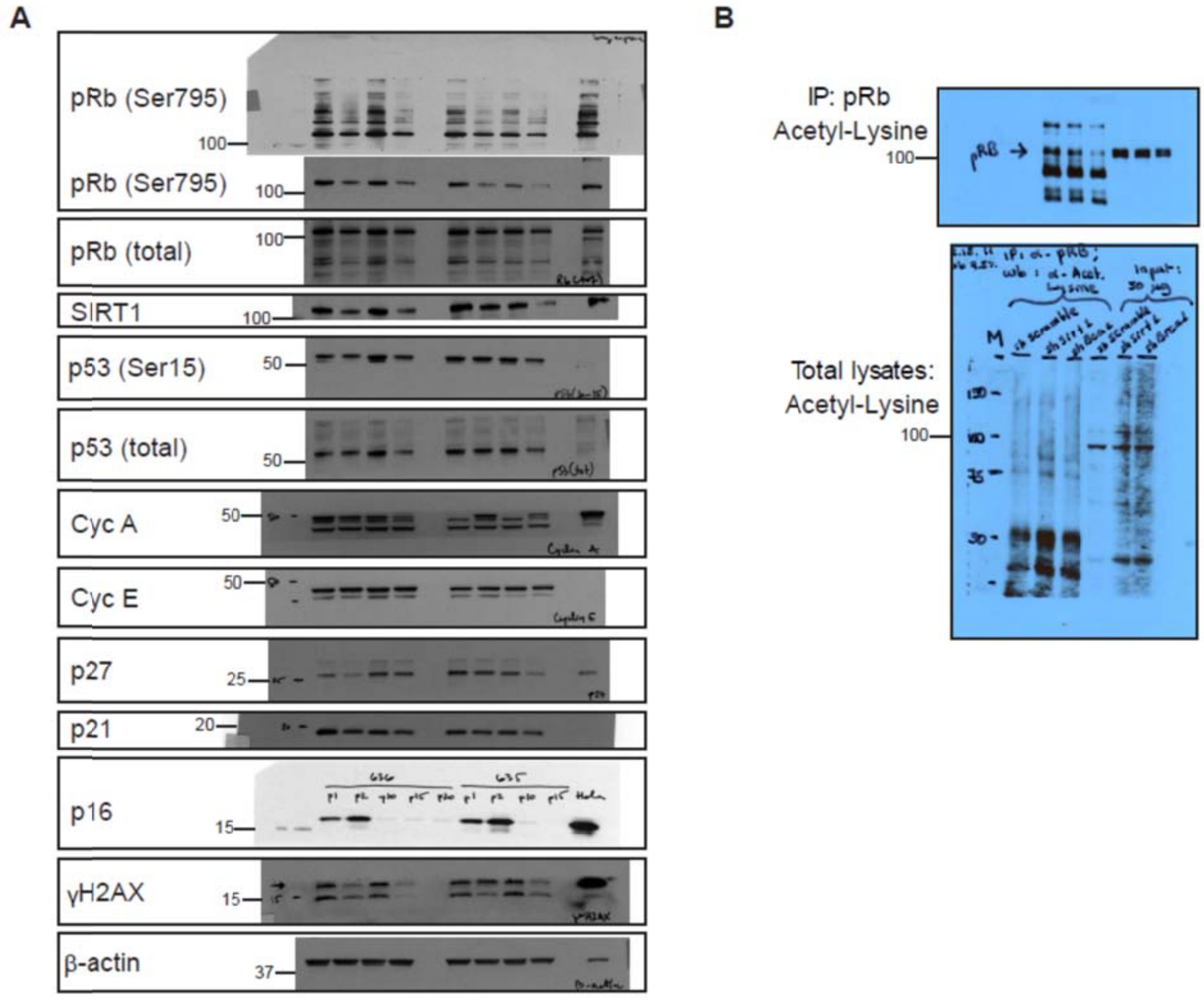

C

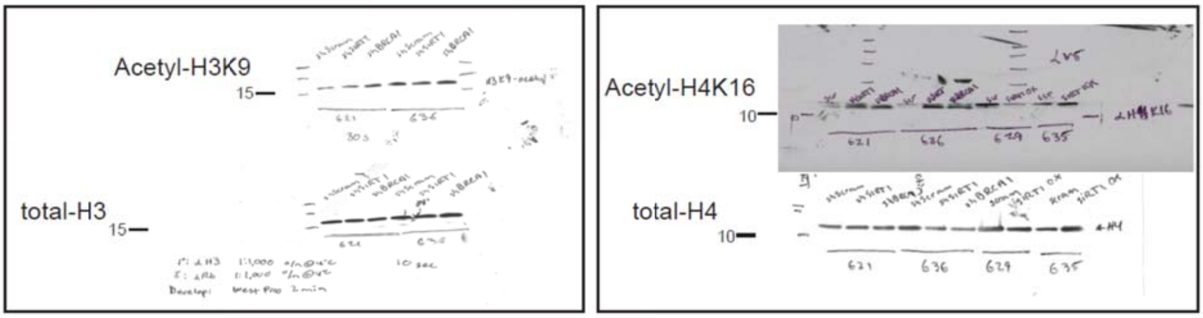

Supplementary Figure 9: Uncropped scans of most relevant westernblots. A) Most relevant blots for pRb (Ser795), pRb (total), SIRT1, p53 (Ser15), p53 (total), Cyclin A, Cyclin E, p27, p21, p16, γH2AX, β-actin. B) pRB IP and total lysates probed with anti-Acetyl-Lysine blots. C) Acetyl-H3K9, total-H3, Acetyl-H4K16 and total-H4 blots.

Supplementary Table 1

Supplemental Table 1. BRCA1 mutation status

Sample ID	Tissue Type	Patient Age	Mutation (y/n)	Specific Mutation	Analysis
616	Proph.Mastec-Brca1	40	yes	exon13ins	qFISH
617	Proph.Mastec-Brca1	53	yes	BRCA1 5385insC	qFISH
627	Proph.Mastec-Brca1	53	yes	BRCA1 187 delAG	qFISH
628	Proph.Mastec-Brca1	40	yes	BRCA1 5385 insC	qFISH
629	Proph.Mastec-Brca1	44	yes	BRCA1 2800 delAA	qFISH, LOH, GC, P/R, TELPCR, MET
634	Proph.Mastec-Brca1	39	yes	BRCA14184del4	qFISH, LOH, TELPCR, MET
635	Proph.Mastec-Brca1	35	yes	BRCA1 5385 insC	qFISH, LOH, GC, P/R, TELPCR, MET
642	Proph.Mastec-Brca1	36	yes	BRCA1 187 delAG	qFISH, LOH, GC, P/R, TELPCR, MET
643	Proph.Mastec-Brca1	47	yes	BRCA1C61G	qFISH
650	Proph.Mastec-Brca1 Skin	33	yes	BRCA1 943ins10	LOH, GC, P/R, TELPCR
651	Proph.Mastec-Brca1 Skin	47	yes	delexon14intron14	GC, P/R, TELPCR
652	Proph.Mastec-Brca1 Skin	46	yes	BRCA1 4154delA	LOH, GC, P/R, TELPCR

qFISH =In vivo telomere length, LOH= LOH analysis; GC= growth curves; P/R= protein and RNA analysis; TELPCR= telomere length PCR
MET=metaphase spreads

Supplementary Table 2

Supplemental Table 2. GSEA analysis					
Name of the pathway	Gene Set SIZE	ES	NES	NOM p-val	FDR q-val
REACTOME_CELL_CYCLE_MITOTIC	261	-0.1822147	-3.333048	0	0
REACTOME_E2F_MEDIATED_REGULATION_OF_DNA_REPLICATION	27	-0.494275	-3.0600283	0	2.95E-04
REACTOME_G2_M_TRANSITION	72	-0.2799382	-2.8140864	0	0.00105462
REACTOME_ACTIVATION_OF_ATR_IN_RESPONSE_TO_REPLICATION_STRESS	33	-0.402977	-2.7753196	0	0.0012494
REACTOME_CENTROSOME_MATURATION	60	-0.2964273	-2.7085989	0	0.00188686
REACTOME_E2F_TRANSCRIPTIONAL_TARGETS_AT_G1_S	18	-0.5194901	-2.6574273	0	0.00293562
REACTOME_G2_M_CHECKPOINTS	38	-0.3559447	-2.5365891	0	0.00567763
REACTOME_ACTIVATION_OF_THE_PRE_REPLICATIVE_COMPLEX	27	-0.3674328	-2.2960405	0	0.01802512
REACTOME_MITOTIC_PROMETAPHASE	71	-0.2303958	-2.2924228	0	0.01653609
BIOCARTA_MCM_PATHWAY	18	-0.4495234	-2.2790813	0	0.01604816
KEGG_CELL_CYCLE	111	-0.1723867	-2.2049823	0	0.02107831
REACTOME_MITOTIC_M_M_G1_PHASES	134	-0.1573849	-2.1053176	0	0.03355169
REACTOME_G1_S_TRANSITION	94	-0.1818364	-2.066683	0	0.03683207
CHICAS_RB1_TARGETS_GROWING	221	0.27887425	4.7940974	0	0
CHICAS_RB1_TARGETS_SENESCENT	495	0.16796239	4.2921963	0	0
KAMMINGA_EZH2_TARGETS	39	0.41060108	3.059856	0	0
V$E2F1_Q3	210	0.15255454	2.5253246	0	2.13E-04
TANG_SENESCENCE_TP53_TARGETS_DN	52	0.2647142	2.2747254	0	0.00351118
CHICAS_RB1_TARGETS_CONFLUENT	490	0.0820208	2.0581293	0.00194932	0.01106058
KUMAMOTO_RESPONSE_TO_NUTLIN_3A_DN	10	0.5047875	1.9616181	0.0020202	0.01498721
REACTOME_DNA_STRAND_ELONGATION	27	-0.2954367	-1.8379503	0.00567108	0.08739737
REACTOME_DNA_REPAIR	93	-0.1684125	-1.8715832	0.01372549	0.07727332
REACTOME_DNA_REPLICATION_PRE_INITIATION	72	-0.1729187	-1.751833	0.01724138	0.1213078
KEGG_HOMOLOGOUS_RECOMBINATION	27	-0.2745898	-1.6734208	0.02249489	0.15364626
REACTOME_SYNTHESIS_OF_DNA	84	-0.1630917	-1.7996148	0.02574257	0.09985348
REACTOME_M_G1_TRANSITION	59	-0.1824439	-1.6782709	0.0308642	0.15378681
REACTOME_CELL_CYCLE_CHECKPOINTS	99	-0.1440138	-1.663237	0.03571429	0.15834156
CHICAS_RB1_TARGETS_LOW_SERUM	76	0.16329645	1.6577407	0.04268293	0.07548876
REACTOME_EXTENSION_OF_TELOMERES	24	-0.2721803	-1.5520241	0.04930966	0.23302579

Supplementary Table 3

shRNA sequences:	
shBRCA1#1:	5’-CCGGGAGTATGCAAACAGCTATAATCTCGAGATTATAGCTGTTTGCATACTCTTTTTG-3’
shBRCA1#2:	5’-CCGGACTGATACTGCTGGGTATAATCTCGAGATTATACCCAGCAGTATCAGTTTTTTG-3’
shSIRT1#1:	5’-CCGGGAGACTGTGATGTCATAATTACTCGAGTAATTATGACATCACAGTCTCTTTTTG-3
shSIRT1#2:	5’-CCGGCAGGTCAAGGGATGGTATTTACTCGAGTAAATACCATCCCTTGACCTGTTTTTG-3’
shpRb#1:	5’-CCGGCCACATTATTTCTAGTCCAAACTCGAGTTTGGACTAGAAATAATGTGGTTTTTG-3’
shpRb#2:	5’-CCGGCAGAGATCGTGTATTGAGATTCTCGAGAATCTCAATACACGATCTCTGTTTTTG-3’

qRT-PCR primers:	
BRCA1 exon4-5:	Forward 5'-GAAACCGTGCCAAAAGACTTC-3'
	Reverse 5'-CCAAGGTTAGAGAGTTGGACAC-3'
BRCA1 exon11:	Forward 5'-TGTGAGTCAGTGTGCAGCAT-3'
	Reverse 5'-CCCAGAGTGGGCAGAGAATG-3'
BRCA1 exon13-15:	Forward 5'- GGAGCAACAGCTGGAAGAGT-3'
	Reverse 5'- GTATGAGCAGCAGCTGGACT-3'
Cyclin A :	Forward 5'-CGCTGGCGGTACTGAAGTC-3'
	Reverse 5'-AAGGAGGAACGGTGACATGC-3'
IL-6 :	Forward 5'-AACCTGAACCTTCCAAAGATGG-3'
	Reverse 5'-TCTGGCTTGTTCCTCACTACT-3'
MMP2 :	Forward 5'-CCGTCGCCCATCATCAAGTT-3'
	Reverse 5'-CTGTCTGGGGCAGTCCAAAG-3'
IL-8 :	Forward 5'-ACTGAGAGTGATTGAGAGTGGAC-3'
	Reverse 5'-AACCCTCTGCACCCAGTTTTC-3'
PAI-1 :	Forward 5'-GCTTGTCCAAGAGTGCATGGT-3'
	Reverse 5'-AGGGCTGGTTCTCGATGGT-3'
Rb :	Forward 5'-GCCTCTCGTCAGGCTTGAG-3'
	Reverse 5'-TCATCTAGGTCAACTCGTGCAA-3'
SIRT1 :	Forward 5'-GCAGATTAGTAGGCGGCTTG-3'
	Reverse 5'-GCTGGTGGAACAATTCCTGT-3'
p14 :	Forward 5'-GGCCCTCGTGCTGATGCTAC-3'
	Reverse 5'-TGGAGCAGCAGCAGCTCCGC-3'
p15 :	Forward 5'-GGACTAGTGGAGAAGGTGCG-3'
	Reverse 5'-GGGCGCTGCCCATCATCATG-3'
p16 :	Forward 5'-CACCGAATAGTTACGGTCGG-3'
	Reverse 5'-GCACGGGTCGGGTGAGAGTG-3'
p18 :	Forward 5'-GGGGACCTAGAGCAACTTAC-3'
	Reverse 5'-GTAGCAGTCTCCTGGCAATC-3'
p19 :	Forward 5'-CTCAACCGCTTCGGCAAGAC-3'
	Reverse 5'-GGACTGGTACCGGAGGTGTC-3'.
GAPDH :	Forward 5'-GAGTCAACGGATTTGGTCGT-3'
	Reverse 5'-TTGATTTTGGAGGGATCTCG-3'

Telomere length qPCR:	
Telg:	ACACTAAGGTTTGGGTTTGGGTTTGGGTTTGGGTTAGTGT
Telc:	TGTTAGGTATCCCTATCCCTATCCCTATCCCTATCCCTAACA
albu:	CGGCGGCGGGCGGCGCGGGCTGGGCGGAAATGCTGCACAGAATCCTTG
albd:	GCCCGGCCCGCCGCGCCCGTCCCGCCGGAAAAGCATGGTCGCCTGTT
hgbu:	CGGCGGCGGGCGGCGCGGGCTGGGCGGCTTCATCCACGTTCACCTTG
hgbd:	GCCCGGCCCGCCGCGCCCGTCCCGCCGGAGGAGAAGTCTGCCGTT

LOH PCR primers:	
BRCA1 187delAG:	Forward 5’-TGTGTTAAAGTTCATTGGAACA-3’
	Reverse 5’-CATAGGAATCCCAAATTAATACA-3’
BRCA1 2800delAA:	Forward 5’- CCACAGTCGGGAAACAAGCATAGA-3’
	Reverse 5’-CACAGGAAAGCCTGCAGTGATA-3’
BRCA1 5385insC:	Forward 5’-AGTCAGAGGAGATGTGGTCAATGG -3’
	Reverse 5’-GTGGTTGGGATGGAAGAGTGAA-3’
BRCA1 4184del4:	Forward 5’-TGGCAAAGGCATCTCAGGAACATC-3’
	Reverse 5’-CTCACTTCTATAAATAGACTGGGGCA-3’
BRCA1 4154delA:	Forward 5’- GCAGTGAATTGGAAGACTTGAC-3’
	Reverse 5’-AAAGCACCTTAGGAGGAACAT-3’
BRCA1 943ins10:	Forward 5’-TCCACCTCCAAGGTGTATGA-3’
	Reverse 5’-TCCTTACTTCCAGCCCATCT-3’
